# The mirror mechanism in schizophrenia: A systematic review and qualitative meta-analysis

**DOI:** 10.3389/fpsyt.2022.884828

**Published:** 2022-09-21

**Authors:** Amir Valizadeh, Mathew Mbwogge, Anita Rasouli Yazdi, Nazanin Hedayati Amlashi, Ainaaz Haadi, Monir Shayestefar, Mana Moassefi

**Affiliations:** ^1^Neuroscience Institute, Tehran University of Medical Sciences, Tehran, Iran; ^2^Independent Researcher, London, United Kingdom; ^3^School of Medicine, Tehran University of Medical Sciences, Tehran, Iran

**Keywords:** mirror neuron activity, schizophrenia, meta-analysis, systematic review, schizophrenia spectrum disorder, mirror neuron system, mirror neurons

## Abstract

**Background:**

Mirror neuron system (MNS) consists of visuomotor neurons that are responsible for the mirror neuron activity (MNA), meaning that each time an individual observes another individual performing an action, these neurons encode that action, and are activated in the observer's cortical motor system. Previous studies report its malfunction in autism, opening doors to investigate the underlying pathophysiology of the disorder in a more elaborate way and coming up with new rehabilitation methods. The study of MNA function in schizophrenia patients has not been as frequent and conclusive as in autism. In this research, we aimed to evaluate the functional integrity of MNA and the microstructural integrity of MNS in schizophrenia patients.

**Methods:**

We included case-control studies that have evaluated MNA in schizophrenia patients compared to healthy controls using a variety of objective assessment tools. In August 2022, we searched Embase, PubMed, and Web of Science for eligible studies. We used an adapted version of the NIH Quality Assessment of Case-Control Studies tool to assess the quality of the included studies. Evidence was analyzed using vote counting methods of the direction of the effect and was tested statistically using the Sign test. Certainty of evidence was assessed using CERQual.

**Results:**

We included 32 studies for the analysis. Statistical tests revealed decreased MNA (*p* = 0.002) in schizophrenia patients. The certainty of the evidence was judged to be moderate. Investigations of heterogeneity revealed a possible relationship between the age and the positive symptoms of participants in the included studies and the direction of the observed effect.

**Discussion:**

This finding contributes to gaining a better understanding of the underlying pathophysiology of the disorder by revealing its possible relation to some of the symptoms in schizophrenia patients, while also highlighting a new commonality with autism.

**Systematic review registration:**

PROSPERO identifier: CRD42021236453.

## Introduction

### Rationale

#### Mirror neuron system; An introduction

The mirror neuron system (MNS), which is a physiological substrate that may subserve certain mechanisms underlying social cognition has recently gained a lot of attention from the research community. MNS is a system consisting of visuomotor neurons that are responsible for the mirror mechanisms, meaning that each time an individual observes another individual performing an action, these neurons which encode that action, are activated in the observer's cortical motor system (1). Observed activations of this system are referred to as mirror neuron activity (MNA). MNA is considered a subdomain of social cognition (2). Several important functions beyond the action domain have been theorized for MNS, such as being a fundamental building block for understanding others' actions (3), encoding the intentions of the actor (4, 5), facilitating imitation (6, 7), and playing a role in human infants' ability to map similarities between self and others (8). Additionally, there has been an emphasis on the possible ties between MNA and empathy (9), and language (10). Mirror neurons were first discovered in the premotor area F5 of macaque monkeys (11). Later, similar neurons were found in the inferior parietal lobule, area PF, of macaque monkeys, and the concept of MNS was established (12). Some studies have claimed the discovery of similar neurons in various regions of the human brain, including the ventral premotor cortex (PMv) (13, 14), inferior frontal gyrus (IFG) (15–17), superior temporal sulcus (STS) (18–20), and inferior parietal lobule (IPL) (14, 21). In the meantime, some counter-arguments exist that question the function and even the very existence of the human MNS, with the strongest argument being the absence of single-cell recording data for human subjects (22). These counter-arguments were assuaged following single-cell recording studies in pre-surgical patients (23), the repetition suppression functional magnetic resonance imaging (fMRI) procedure in healthy volunteers (17), and lesion study in the human brain regions that have been proposed to be associated with human MNS (24). Nevertheless, MNS has been one of the most widely investigated domains of social cognition in psychiatric disorders within human beings. Even a recent paper by Heyes and Catmur (25) has called for more research on this phenomenon.

Other regions have also been proposed as an extension to MNS, one of the most important of them being the Brodmann area 2 (BA2) (26), which is the strongest generator of the mu rhythm (27). Mu rhythm (oscillations from 8 to 13 Hz) suppression has been proposed to be an indication of the MNA, as it is seen both when an individual performs and observes an action (28–30). A meta-analysis has demonstrated that there might also be other brain regions that do not have mirror properties but may convey necessary information to MNS including the primary visual cortex, supplementary motor area, dorsal premotor cortex, superior parietal lobe, cerebellum, and parts of the limbic system (31).

#### MNA in psychological disorders; Broken mirror theory and autism

In the context of psychological disorders, MNA has been mostly investigated in autism. This is due to the “broken mirror” hypothesis and its role in explaining the social and language deficits of this disorder (32). However, research has produced insufficient evidence to support this hypothesis in its pure form, and instead, two alternative models have been proposed: the EP-M model and the social top-down response modulation (STORM) model. The STORM model proposes that autism symptoms originate from abnormalities within the top-down regulation of the MNS, rather than within the MNS itself, while the EP-M model proposes that imitation behavior in autistic individuals is served by the pathways between brain areas associated with MNS (33). Nevertheless, both these alternative models also suggest that there might be some possible dysfunction in the MNA in these patients, either within the MNS itself or within the systems that regulate MNA (32, 33). The discovery of such dysfunction has opened the doors to investigate and explain the underlying pathophysiology of the disorder in a more elaborate way and to come up with new rehabilitation methods (34–36).

#### MNA dysfunction in schizophrenia and autism; A commonality?

Schizophrenia is one of the most debilitating and common neuropsychiatric disorders, with an estimated prevalence between 0.28 and 0.75% in the population worldwide (37–40). Deficits in a variety of cognitive domains are well-known for this disorder, such as impaired attention, verbal memory, and social cognition, and these are listed as specifiers for schizophrenia in the 11th revision of the International Classification of Diseases (ICD-11) (41). There are several reports of individuals with both autism and schizophrenia (42–45), which reveal that deficits in the theory of mind (ToM) exist in both disorders. Also, there are reports that both disorders share several genetic signals (46). A previous meta-analysis (47) of fMRI studies on autism and schizophrenia patients during ToM tasks revealed hypoactivation in the STS area, one of the main brain regions associated with MNA, in both groups, yet again emphasizing the deficits in ToM in both disorders, and possibly, hypothesizing the presence of MNA impairments in schizophrenia similar to the already known MNA impairments in autism patients.

MNA dysfunction might be another commonality between these disorders. Investigations of MNA in schizophrenia have not been as profound and conclusive as in autism. Based on a recent review (48) that partly examined this subject, findings of the state of MNA function in schizophrenia are mixed, with some studies suggesting impaired MNA function in the patients, while others did not find such a phenomenon. If such dysfunction is proven to be present in schizophrenia patients, it might potentially serve for implementing new rehabilitation treatments based on MNA training, as such treatment options have been previously found in multiple reports to be beneficial for autism patients (49–53).

### Objectives

To date, there has not been a systematic review with a qualitative analysis of studies that examine MNA/MNS in schizophrenia patients. Although a previous systematic review of the studies exists (54), that paper is a review of the evidence with little data analysis, and thus, considering the importance of the functions theorized to be associated with MNA, and the new studies published since that review, a new systematic examination of studies on this subject with a more in-depth analysis of the findings seems necessary. Results of such investigations may also help in gaining a more in-depth understanding of the mechanisms underlying schizophrenia and its possible common pathogenesis with autism. Specifically, we aim to evaluate the following:

- Primary objective: The functional integrity of MNA in schizophrenia patients. By functional integrity, we mean evaluating MNA using brain function measurement methods to investigate if it is identical to those in healthy control subjects.
- Secondary objective: The microstructural integrity of MNS in schizophrenia patients. By microstructural integrity, we mean evaluating MNS using brain microstructure evaluation methods to investigate if it is identical to those in healthy control subjects.

## Methods

The design and methods used for this review comply with the Center for Reviews and Dissemination (CRD) Guidance for Undertaking Reviews in Healthcare (55), a guideline that presents rigor methods for undertaking systematic reviews, and Meta-analyses of Observational Studies in Epidemiology (MOOSE) (56), a guide on methods for conducting systematic reviews and meta-analyses specifically on observational studies. This review is reported in line with Preferred Reporting Items for Systematic Reviews and Meta-Analyses (PRISMA) (57) guideline. The review protocol has been published elsewhere (58).

### Eligibility criteria

Eligibility criteria for including studies were informed using the SPIDER (Sample, Phenomenon of Interest, Design, Evaluation, Research type) framework (59):

**Sample:** Patients of any age and sex diagnosed with schizophrenia or schizoaffective disorder confirmed by a physician in line with the International Classification of Diseases (ICD) or Diagnostic and Statistical Manual of Mental Disorders (DSM), irrespective of the severity or duration of illness, compared to healthy controls. Participants with any other macrostructural or functional neurologic disorders were excluded.

**Phenomenon of interest:** MNA and microstructural integrity of main brain regions (PMv, IFG, STS, IPL, and BA2) that are theorized to be associated with MNS.

**Design:** Observational case-control studies.

**Evaluation:**

- Functional methods: Electroencephalography (EEG), Magnetoencephalography (MEG), Transcranial magnetic stimulation (TMS), Electromyography (EMG), Proton Emission Tomography (PET), and Functional magnetic resonance imaging (fMRI). These methods are indirect measurements of what may reflect MNA, based on prior literature, as we cannot directly measure MNA in humans yet.
- Microstructural methods: Diffusion Tensor Imaging (DTI), Diffusion-Weighted Imaging (DWI), and Diffusion Spectrum Imaging (DSI). Only studies that specifically aimed to evaluate the microstructural integrity of the MNS were included.

**Research type:** Qualitative, quantitative, and mixed-methods.

### Information sources and search strategy

In August 2022, AV searched Embase (*via* Ovid), PubMed, and Web of Science for eligible studies. We also carried out a “snowball” search through forward-citation and backward-citation tracking using Scopus on all of the included studies. Our search strategy is reported in line with the Preferred Reporting Items for Systematic Reviews and Meta-Analyses literature search extension (PRISMA-S) (60). No restriction or search filter was used. The search strategy is presented in Data sheet 1.

### Selection process

Records were imported to EndNote version X9. NH and AH independently reviewed the titles and abstracts of the retrieved records. AV was consulted to make the final decision in cases of disagreements. The full texts of all potentially eligible records were retrieved. MMb and AR independently screened full-text studies. A study was included when both reviewers independently assessed it as satisfying the inclusion criteria.

### Data collection process

A data extraction form was developed, pilot tested, and then refined. After finalizing the data extraction form, MMb, AR, and MSh independently used it to extract data from eligible studies. Extracted data were compared, with any discrepancies being resolved through discussion. AV entered data into Microsoft Excel, double-checking them for accuracy. When information regarding any data was unclear, we contacted the authors of the reports to provide further details.

### Data items

We extracted the following information from the included studies:

- Sample size and characteristics such as age, gender, handedness, and ethnicity;
- Inclusion and exclusion criteria of the study;
- Assessment tool information (paradigm class and equipment properties);
- Ethical considerations;
- Severity score of the disease;
- Brain regions with different activation patterns between patients and controls (for task-based fMRI, MEG, and PET);
- Results and conclusions of the study; and
- Funding sources and conflicts of interest.

Ethnicities were categorized according to the NIH Racial and Ethnic Categories (61). Severity scores included the Positive and Negative Syndrome Scale (PANSS) (62), the Scale for the Assessment of Negative Symptoms (SANS) (63), and the Scale for the Assessment of Positive Symptoms (SAPS) (64). To achieve a better comparison state, SANS and SAPS were converted to PANSS (65) scores.

### Study methodological and reporting quality assessment

We used an adapted version of the NIH Quality Assessment Tool for Case-Control Studies (66). This tool is originally developed to evaluate the internal validity of case-control studies, is consisted of 11 questions, and assesses the following factors: risk of potential for selection bias, information bias, measurement bias, confounding, exposures occurring before outcomes, evaluation of a dose-response gradient, accuracy of measurement of both exposure and outcome, sufficient time frame to see an effect, and appropriate control for confounding. Using this tool, the overall methodological and reporting quality of a study should be judged as either poor, fair, or good.

After consensus, we made some changes to the tool, so it better suits our review. As sample size justification does not apply to our topic, we changed the third question to check if the authors included a considerable sample size. Considering the multimodal nature of this review, it was not possible to use power analysis to calculate the minimum required sample size for each study. Considering a recent analytical study (67), a sample size of 34 participants is required to surpass 80% power to detect an effect size of D = 0.5 at α = 0.05 (though usually in functional neuroimaging studies α = 0.001 is the standard). Nevertheless, investigations revealed that 90% of the highly cited fMRI papers had a sample size smaller than that (67). Considering these facts, by consensus, we decided to define the minimum required sample size as at least 34 participants (17 for each group). We changed the fourth question to address one of the most important possible confounders in our review, unrelated concurrent psychiatric and neurologic disorders. We considered the minimum required inclusion/exclusion criteria to address substance dependence, and other possible medical disorders, and having specified the diagnostic criteria used to diagnose patients. Also, as the 8th and 9th (concurrent control and exposures occurring before outcomes) questions don't apply to our subject, we changed them to address if controls were matched with cases for age, gender, and handedness because they might be important confounders in our study. We defined matching for age as having a *p* > 0.05 for the difference between groups, while for gender, we defined it as having a *p* > 0.5. We modified the 10th question to assess the validity and sufficient report of the paradigm used in the study. We considered a paradigm valid if at least some methodological studies have previously confirmed its reliability for the assessment of MNA. In the case of methodological innovations, the validity of the paradigm was assessed subjectively by discussion among the reviewers. Also, in cases of the inadequate report of paradigm parameters (e.g., not reporting acquisition parameters of an fMRI experiment), the study was ranked poor for this domain. The reviewers' arguments for each subjective decision behind the validity and report of the paradigms used in each study are presented in detail in Data sheet 2. We also removed the 11th question which addressed blinding of outcome assessors, since interventional methods (where blinding is of paramount concern) do not apply to our subject. Finally, we changed the last question to check if ethical issues were considered in the study design.

The adapted version of the tool was pilot-tested before use. MMb and AR independently evaluated included studies and recorded supporting information and justifications for their judgments. In cases of disagreements, AV was consulted.

### Analysis methods

#### Eligibility and preparing for analysis

As we included data from multiple paradigms, with different outcomes, quantitative analysis was not feasible. Thus, we aimed for qualitative analysis. The full texts of the included studies were read and evaluated by AV and MMo. We determined the direction of the effect based on the studies' results, as either “decreased,” “intact,” or “increased MNA” for the primary outcome and “intact” or “altered MNS” for the secondary outcome. Regarding the primary outcome, we only included studies that have directly evaluated MNA. We did not consider studies that assessed other cognitive domains hypothesized to be related to MNA (e.g., empathy, etc.) as eligible for analysis. For the secondary outcome, we did not perform any analysis as there were very few studies for this purpose.

#### Statistical analyses

AV analyzed the data using Microsoft Excel and dmetar (68) package for R version 4. A qualitative meta-analysis was performed for the primary outcome based on vote counting of the direction of the effect. Vote counting, a simple method for analyzing evidence from multiple evaluations, involves comparing the number of studies showing benefit (reduced MNA in the case of our study) with the number of studies showing harm (intact/increased MNA in the case of our study) (69). A harvest plot was designed to present results from the analysis. We also designed graphics to represent evaluated domains of methodological and reporting quality for each study and the quality across all studies.

To test for the statistical significance of the vote counting analyses, we used the sign test. The sign test is a non-parametric test that uses a binary measure of either a positive or a negative effect to test whether there is sufficient evidence to reject the null hypothesis of an equal number of positive and negative results (70). The *P*-value from a sign test represents the probability of observing the given number of positive and negative results if the null hypothesis was true. To perform the test, we counted the number of studies in each effect direction for the outcome. Also, to explore the results of the most commonly used paradigms, we conducted separate analyses on paradigms with more than 5 studies, which were EEG with 7 studies and task-based fMRI with 9 studies. We used GraphPad (Link) to calculate the two-tailed *P*-value for the sign test. We considered a *p* < 0.05 as significant (alpha error).

#### Subgroup analyses

To explore heterogeneity in the results, we compared the outcome between subgroups. We conducted a test for subgroup differences between studies that evaluated MNA in “drug-naïve/drug-free for at least 1 month” patients, against studies on “medicated” patients. To check for this difference, we conducted Fisher's exact test (Link). Also, knowing that gray matter volumes atypically decline with age in schizophrenia patients (71), we conducted a logistic meta-regression test by comparing the mean age of the participants in each study, against the direction of the effect. We used the weighted least squares (WLS) method for this regression, with the weight associated with each study being the square root of its sample size ($\sqrt{N}$). Similar subgroup analyses were done for the gender of the participants (female to male ratio), mean positive PANSS scores of patients, and mean negative PANSS scores of the patients against the direction of the effect.

#### Sensitivity analyses

To evaluate the robustness of our results, we conducted a sensitivity analysis by excluding studies that were judged to be of poor methodological and reporting quality. We used the same previous methods above for this analysis.

### Certainty assessment

The strength of the overall body of evidence was assessed using the Confidence in Evidence from Reviews of Qualitative Research Methods (CERQual) (72). This approach evaluates four components to score confidence in the review findings. These include methodological limitations, relevance, coherence, and adequacy. Each finding starts with a “high confidence” score which could be downgraded to “moderate confidence,” “low confidence,” or “very low confidence” if the CERQual process revealed concerns. AV and MMo evaluated each finding using the tool and attributed a score to it based on the four-point scoring system. We resolved discrepancies through discussion.

## Results

### Study selection

**Figure 1:**
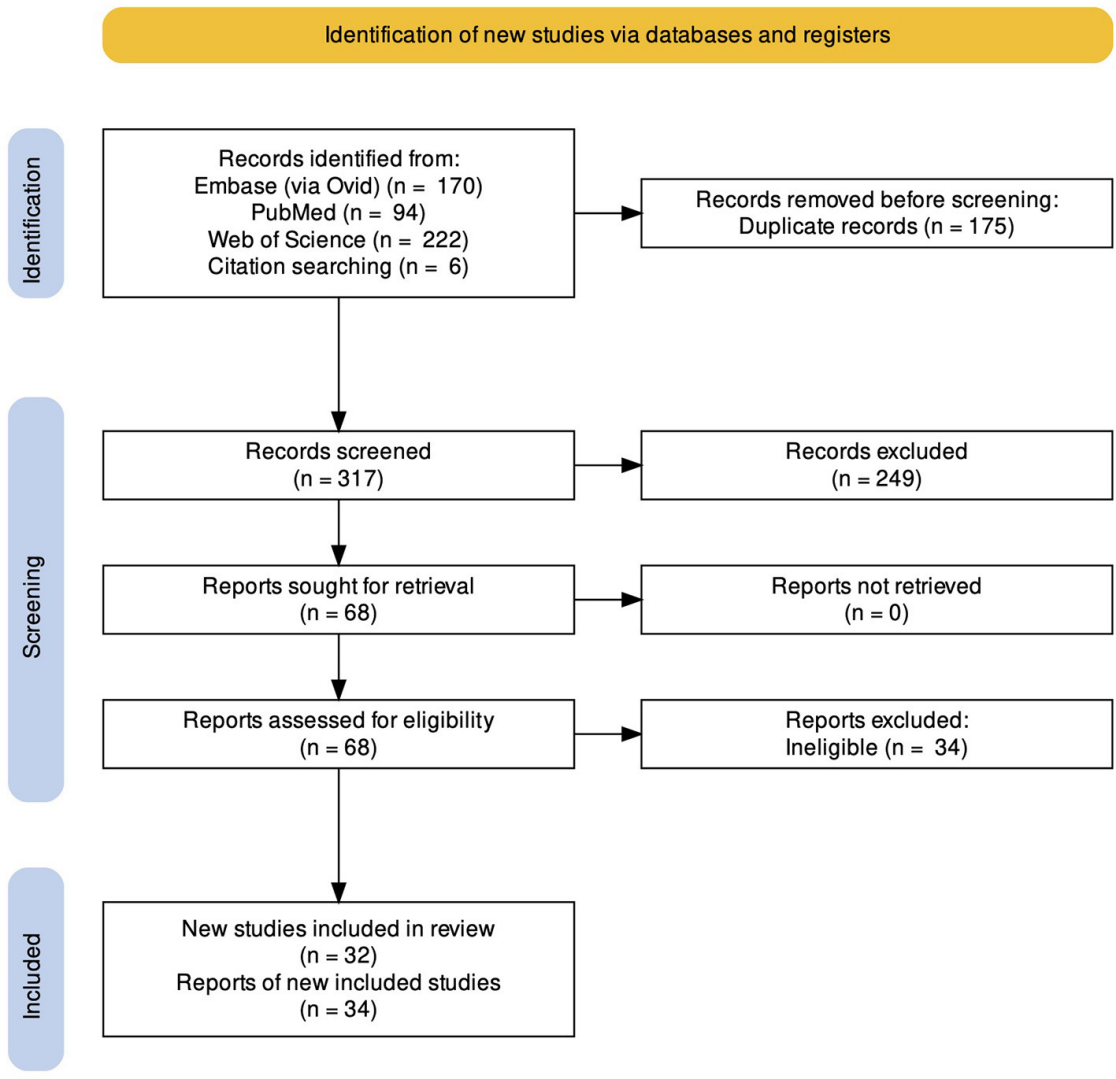
PRISMA Flow diagram of the study. We identified 486 records through database searching and six records through citation searching. Following deduplication, 317 records were screened, from which, 32 relevant studies (34 reports) were found and included in the review.

We identified 486 records through database searching. After deduplication and screening titles and abstracts of the records, 424 records were excluded. After reviewing the full texts of these reports, 28 were found to be eligible for inclusion in the review. Following citation searching of these studies, 6 more eligible studies were found. In the end, 32 studies (34 reports) were included in this review, 29 for the main outcome (functional integrity of MNA) and 3 for the secondary outcome (microstructural integrity of MNS). A detailed report of the study selection process is presented in Figure 1. It is of special notice that the three papers of Horan et al. were considered as one study for the statistical analyses (since they were performed on the same patients in the same setting), but were assessed for methodological quality separately (because they reported three different phases of a study).

### Study characteristics

We included 29 studies (73–103) with 1,542 participants for the primary outcome and 3 studies (104–106) with 126 participants for the secondary outcome. Overall, 32 studies were included in this systematic review. For a detailed summary of the characteristics of the included studies, see Data sheet 3.

### Methodological and reporting quality of studies

**Figure 2:**
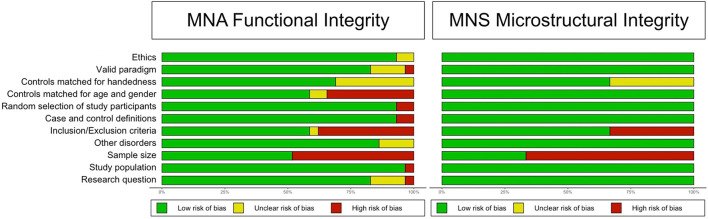
Methodological and reporting quality graph: Review authors' judgments about each methodological and reporting quality item presented as percentages across all included studies.

**Figure 3:**
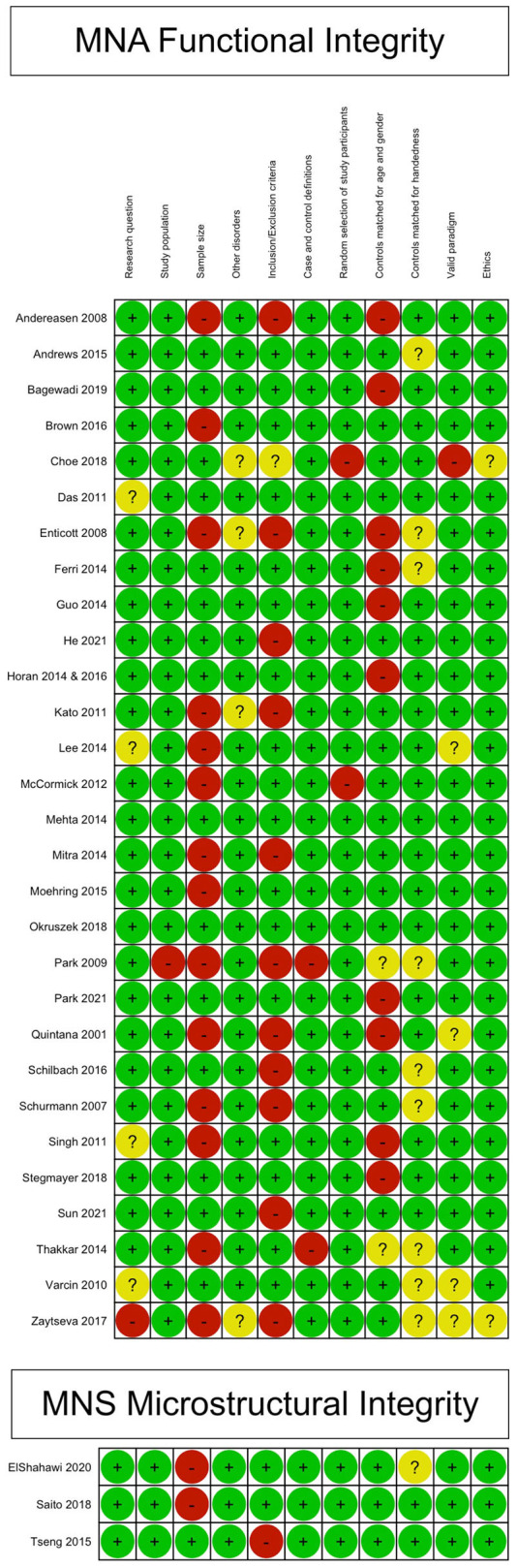
Methodological and reporting quality summary: review authors' judgments about each methodological and reporting quality item for each included study.

Sixteen studies (14 for the primary outcome, 2 for the secondary outcome) were judged to have good methodological and reporting quality, eight (7 for the primary outcome, 1 for the secondary outcome) were judged to have fair quality, and ten (all for the primary outcome) were judged to have poor quality. For more information on the quality domains for each study, please check Data sheets 2, 3. Figure 2 shows the judgments for each domain in each included study for each outcome. Judgments for each domain and each outcome across all studies are presented in Figure 3.

### Results of individual studies

**Figure 4:**
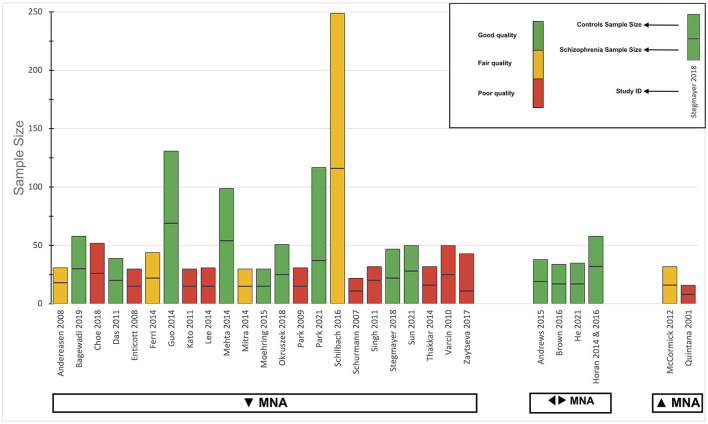
Harvest plot of the overall analysis for the primary outcome. The height of each bar represents the sample size, divided by a line into two sections to represent the sample size of each group (case and control). The methodological and reporting quality of each study is presented by the color of the bar; green for good, yellow for fair, and red for poor. The direction of the effect for the studies is mentioned below the bars: ▾ for decreased MNA, ◂▸ for intact MNA, and ▴ for increased MNA. MNA, Mirror neuron activity.

Regarding the primary outcome, the direction of the effect in most studies was toward decreased MNA. Four studies concluded that MNA in schizophrenia patients was not different from healthy controls, while two studies indicated that they detected increased MNA in these patients. For a detailed summary of the results of individual studies for this outcome, see the harvest plot in Figure 4. All three studies that evaluated the secondary outcome concluded that MNS microstructural integrity was altered in patients.

### Results of analyses

#### Characteristics of contributing studies

A summary of the characteristics of the included studies is presented in Table 1. The comments section for this table is built upon the comments provided in a similar table in the systematic review of Mehta et al. (54). For a more detailed report of the characteristics of contributing studies, see Data sheet 3.

**Table 1:** The effect direction of the outcome table.

**References**	**Direction**	**N (SCZ/HC)**	**Mean age**	**Medicated**	**+PANSS**	**–PANSS**	**Setting**	**Paradigm**	**Experimental condition**
**Microstructural methods**									
Tseng et al. (106)	Altered	32/32	32	✓	–	–	Inpatient	DSI	Microstructural data
ElShahawi et al. (104)	Altered	15/15	29	✓	–	–	Mixed	DWI/DTI	Microstructural data
Saito et al. (105)	Altered	16/16	21	✓	–	–	Mixed	DWI/DTI	Microstructural data
**Functional methods**									
Brown et al. (76)	**◂▸**	17/17	40	✓	19 (7)	25 (8)	Inpatient	EEG	(a) Rest: inanimate motion, (b) Action-observation: observing video clips of two people sitting at a table, transferring coins from one bowl to the other bowls at the table
Horan et al. (84)	**◂▸**	32/26	46	✓	–	–	Outpatient	EEG	(a) Rest: inanimate motion (two bouncing balls), (b) Action-observation: hand movements, people playing a throw and catch game by throwing a ball to themselves, to each other, and to and from the observer
McCormick et al. (87)	**▴**	16/16	37	✓	17 (12)	16 (10)	Inpatient	EEG	(a) Rest: watching snow-fall, (b) Action-observation: bouncing balls and hand movements
Mitra et al. (89)	**▾**	15/15	29	×	–	–	Inpatient	EEG	(a) Rest: White screen, (b) Action-observation: video of handshakes, repeated at a rate of 1 per second
Möhring et al. (90)	**▾**	15/15	35	✓	16 (4)	20 (5)	Outpatient	EEG	(a) Action-observation: observing a static image of gestures of a hand for the rock–paper–scissors game, (b) Action-execution: participants actively executed hand gestures when stimuli depicting rock, paper, or scissors were displayed
Singh et al. (95)	**▾**	20/12	21	✓	15 (15)	17 (13)	Outpatient	EEG	(a) Rest: inanimate motion (two bouncing balls), (b) Action-observation: hand movements, point light display animation of a jumping human, people playing a game of throw and catch
Zaytseva et al. (101)	**▾**	11/32	23	✓	–	–	–	EEG	Imaginary representation of one's own walking on a familiar street (2 min) followed by the subjects' self-reports
Varcin et al. (99)	**▾**	25/25	42	✓	15 (13)	16 (10)	Outpatient	EMG	Watching facial expressions of happiness and anger displayed in 4 male and 4 female faces, while EMG was recorded from zygomaticus major and corrugator supercilii
Das et al. (78)	**▾**	20/19	34	✓	10 (3)	18 (5)	Inpatient	fMRI	16 blocks: 8 experimental in which two triangles mimicked human behavior (bluffing, persuading, surprising, and mocking), and 8 controls in which two triangles moved randomly
Ferri et al. (80)	**▾**	22/22	28	≈	14 (4)	12 (5)	Outpatient	fMRI	336 trials where subjects watched either “emotion action,” “emotion,” or “action” stimuli and 32 imitation trials where subjects were given a request to imitate either the action or the emotion
He et al. (102)	**◂▸**	17/18	32	✓	26 (17)	16 (13)	Inpatient	fMRI	Two runs of 182 trials each. Each run consisted of 3 stimuli: (a) observing videos of an actor making incomprehensible Russian sentences with gestures, (b) making comprehensible German sentences without any gestures, (c) making German sentences with accompanying gestures
Horan et al. (82)	**◂▸**	23/23	47	✓	–	–	Outpatient	fMRI	Five runs of 6 blocks, each block consisted of 6 trials (3 fingers and 3 faces). The trials required subjects to either (a) observe: observe finger movements or a facial expression, (b) imitate: imitate the fingers movement or the facial expression, and (c) execute: make the movement or facial expression described by each word. Words included the following in a random order: Lift Index, Lift Middle, Happy, Sad, Angry, Afraid
Horan et al. (83)	**◂▸**	21/21	47	✓	–	–	Outpatient	fMRI	Four runs of a mixed blocked/event-related paradigm. Each run consisted of two components: (a): (i) observing videos of patients receiving a painful sound stimulation treatment; (ii) listening to the painful sounds (to create ROIs). (b): manipulations of perspective-taking (imagine “Self” vs. “Other” experiencing pain) and cognitive appraisal (treatment was “Effective” vs. “Not Effective”)
Lee et al. (86)	**▾**	15/16	37	✓	10 (3)	13 (3)	Outpatient	fMRI	180-trials (0.5 s of watching phase for each); (a) observation phase: subjects watched either facial or word stimuli, (b) expression phase: subjects actively expressed the emotions displayed, (c) returning phase: subjects returned to neutral facial expression after watching a neutral cue on the screen
Okruszek et al. (100)	**▾**	25/26	35	✓	11 (3)	18 (4)	Outpatient	fMRI	112 trials–each trial consisted of (a) watching phase: watching animations displaying actions of agents presented as point-light walkers, (b) behavioral response phase: responding to the question “Are the two persons acting together or separately?,” (c) ISI phase
Park et al. (91)	**▾**	15/16	–	✓	13 (2)	17 (4)	Outpatient	fMRI	24 blocks; each block consisted of perceiving, inferring, and selecting appropriate responses (30, 20, and 10 s, respectively), to ambiguous or certain emotional events narrated by a graphical avatar. The neutral certain condition was the control condition
Quintana (92)	**▴**	8/8	33	✓	–	–	Outpatient	fMRI	Four runs of block-design paradigms–each run consisted of 3 resting blocks scattered among 2 sets (colored circles or drawings of facial expressions) of 6 task trials, where the subject was required to match the cues
Stegmayer et al. (96)	**▾**	22/25	38	✓	18 (7)	19 (5)	Mixed	fMRI	Two runs of event-related paradigm–each run consisted of 3 phases: (a) visual command phase (3 s), (b) planning phase (3 s): participants had to plan movements, (c) execution phase (3 s): participants should've executed the gesture that was stated in the visual command phase
Thakkar et al. (98)	**▾**	16/16	39	≈	14 (10)	23 (12)	Inpatient	fMRI	Four runs of 14 blocks–each block consisted of 3 trials (3 movement conditions in each). Subjects were required to either execute actions of pressing buttons while viewing these stimuli or observe (a) a hand pressing buttons, (b) an image of a hand and a button box, (c) inanimate marks
Kato et al. (85)	**▾**	15/15	33	×	18 (4)	18 (8)	-	MEG	(a) Rest: eyes fixed on a cross, (b) Action-observation: mouth opening movements.
Schürmann et al. (94)	**▾**	11/11	54	✓	-	-	Outpatient	MEG	(a) Rest: resting in a relaxed state, (b) Action-observation: manipulation of a small object with a hand; (c) Action-execution: participants manipulated the small object with their hand
Andereasen et al. (73)	**▾**	18/13	30	×	12 (11)	9 (8)	Outpatient	PET	Subjects were asked to say narrative stories explaining a given social situation. The control task required subjects to read aloud a neutral story that was presented on the monitor
Choe et al. (77)	**▾**	26/26	23	≈	16 (4)	16 (4)	Outpatient	rs-fMRI	Resting-State
Guo et al. (81)	**▾**	69/62	31	✓	12 (5)	14 (6)	Inpatient	rs-fMRI	Resting-State
Park et al. (103)	**▾**	37/80	23	≈	16 (4)	17 (5)	Outpatient	rs-fMRI	Resting-State
Schilbach et al. (93)	**▾**	116/133	34	✓	–	–	Multi-centric	rs-fMRI	Resting-State
Sun et al. (97)	**▾**	28/22	17	×	23 (7)	17 (7)	Inpatient	rs-fMRI	Resting-State
Bagewadi et al. (75)	**▾**	30/28	27	✓	21 (16)	20 (16)	Inpatient	TMS	(a) Rest: observing a static image, (b) Natural action-observation: a key held in pinch grasp, performing locking and unlocking, (c) Context-based action-observation: observing a video clip of a mother trying to unlock the door of a house that is on fire and her child is stuck in calling for help
Enticott et al. (79)	**▾**	15/15	38	✓	15 (4)	15 (5)	-	TMS	(a) Rest: not specified, (b) Action-observation: non-goal directed and goal-directed finger movements
Mehta et al. (88)	**▾**	54/45	31	≈	24 (6)	23 (9)	Mixed	TMS	(a) Rest: observing a static image, (b) Action-observation: a key held in pinch grasp, performing locking, and unlocking movements
Andrews et al. (74)	**◂▸**	19/19	41.0	✓	16 (6)	16 (5)	Outpatient	TMS/EEG	(a) Rest: observing a black screen, (b) Action-observation: 6 video clips: 2 static hands; a hand reaching out and clasping a mug; a hand pantomiming clasping a mug; and 2 interactive movements, one with hands from two different people, and a similar movement carried out by one person

▴ indicates increased mirror neuron activity (MNA) ◂▸ indicates intact MNA, and ▾ indicates decreased MNA. Colors represent the methodological quality of the study (green = good, yellow = fair, red = poor). ✓, Mostly medicated patients; × , Mostly drug-naïve/drug-free for at least a month; ≈, Mixed. + PANSS: + Positive and Negative Syndrome Scale (PANSS) scores [mean (SD)] round to the nearest integer; –PANSS, –PANSS scores [mean (SD)] round to the nearest integer; DSI, Diffusion spectrum imaging; DTI, Diffusion tensor imaging; DWI, Diffusion-weighted imaging; EEG, Electroencephalography; EMG, Electromyography; fMRI, Functional magnetic resonance imaging; HC, Healthy controls; MEG, Magnetoencephalography; PET, Proton emission tomography; rs-fMRI, Resting-state fMRI; SCZ, Schizophrenia; TMS, Transcranial magnetic stimulation.

#### Patterns of activity in MNA-specific brain regions

MEG, fMRI, and PET are known to provide good spatial resolutions. The different patterns of activity in MNA-specific brain regions between cases and controls in the included studies are provided in Table 2.

**Table 2:** The difference in the pattern of activation of different mirror neuron activity (MNA)-specific brain regions between schizophrenia and healthy control participants in task-based fMRI, MEG, and PET studies of MNA.

**References**	**Modality**	**PMv**	**IFG**	**IPL**	**STG**	**Insula**
Andereasen et al. (73)	PET	–	Lower	–	–	–
Das et al. (78)	Task-Based fMRI	–	Lower	Lower	Lower	–
Ferri et al. (80)	Task-Based fMRI	–	Lower	Lower	–	Lower
Kato et al. (34)	MEG	–	-	Lower	–	-
Lee et al. (35)	Task-Based fMRI	Lower	Lower	Higher	–	Higher
Okruszek et al. (100)	Task-Based fMRI	–	–	–	Lower	–
Park et al. (91)	Task-Based fMRI	Lower	Lower	–	–	–
Quintana (92)	Task-Based fMRI	Higher	Higher	–	–	–
Schurmann et al. (94)	MEG	Lower	–	–	–	–
Stegmayer et al. (96)	Task-Based fMRI	–	Lower	Higher	–	–
Thakkar et al. (98)	Task-Based fMRI	–	–	Higher	Lower	–

Lower means lower activity in patients compared to controls, while higher means higher activity. IFG, Inferior frontal gyrus; IPL, Inferior parietal lobule; STG, Superior temporal gyrus; PMv, Ventral premotor cortex.

IFG was the most investigated area across the literature where 6/7 studies reported the detection of a decreased MNA in that region. IPL was the second most investigated area, but interestingly, it was also the one with the most controversial results. Of the 6 studies that evaluated this area, 3 reported the detection of decreased MNA and the other 3 reported the detection of increased MNA. Also, MNA was reported to be decreased in PMv in 3 of the 4 studies that investigated this area. Results for the STG area were pretty consistent with 3 of 3 studies reporting decreased MNA. Only 2 studies reported a difference in the insula activation, where their results were in the opposite direction.

#### Results of statistical analyses

The results of the analysis for the primary outcome are presented as a harvest plot in Figure 4. Most studies concluded that MNA was significantly reduced in schizophrenia patients, compared to controls (23/29, 79.3%). The two-tailed sign test *P*-value was calculated to be 0.002, meaning that the chance of observing either 23 or more studies, or 6 or fewer studies in 29 studies, in that direction, is 0.2%. Only two studies (87, 92) found significant results in the opposite direction (2/29, 7.9%). Four studies (74, 75, 82–84, 102) concluded that there was no significant difference between patients and healthy controls (4/29, 13.8%).

We also conducted a vote-counting analysis for the direction of the effect for studies that only used task-based fMRI as their assessment tool. In this group, seven studies concluded that MNA was reduced in cases, although this finding was not statistically significant (7/10, 70.0%; *P* = 0.344). A similar analysis was also conducted for studies that only used EEG. In this group, four studies concluded that MNA was reduced in cases (4/7, 57.1%; *P* = 1.000), showing almost no statistical significance. Also, two studies showed an intact MNA and one concluded that MNA was increased in cases. Results were very contradictory for the EEG group and demanded explicit evaluation.

#### Results of subgroup analyses

Regarding the primary outcome, for the patients in the “drug-naïve/free for at least 1 month” subgroup, 4/4 studies, and the patients in the “medicated” subgroup, 14/20 studies were in the direction of decreased MNA, while 6 studies were in the direction of either intact or increased MNA. The test for subgroup differences revealed no significant difference between them (*P* = 0.539).

**Figure 5:**
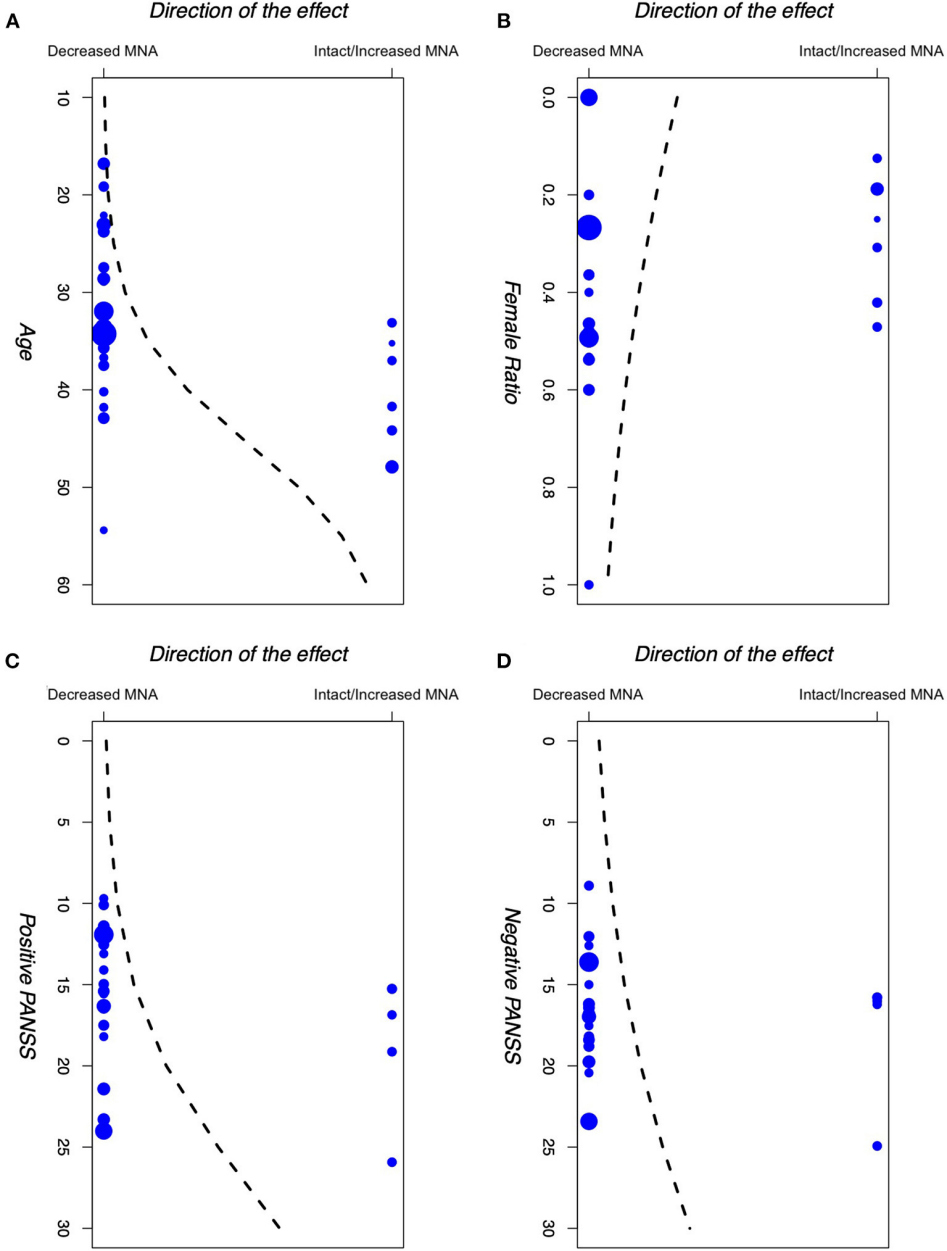
Logistic meta-regression analyses for **(A)** age, **(B)** gender (female to male ratio), **(C)** +PANSS scores, and **(D)** −PANSS scores of participants in the included studies against the direction of the effect of those studies. PANSS: Positive and Negative Syndrome Scale.

Results for the logistic meta-regression analyses for age, gender (female to male ratio), positive PANSS scores, and negative PANSS scores against the direction of the effect for the primary outcome are presented in Table 3 and Figure 5. There seems to be a relationship between the age of participants and the direction of the effect. A similar relationship was observed between the positive PANSS scores and the direction of the effect. Studies that found intact/increased MNA, were performed on patients of higher age and higher positive PANSS scores. These relationships were found to be statistically significant (*P* < 0.001 for age and *P* = 0.004 for positive PANSS scores).

**Table 3:** Results of the logistic meta-regression analyses for investigating the possible causes of heterogeneity.

**Dependent variable**	**Independent variable**	**β_0_ (Intercept)**	**β_1_**	***p*-value**
Direction of	Age	−7.42	0.16	< 0.001
the effect	Female to male ratio	−0.82	−1.86	0.070
	Positive PANSS	−4.75	0.17	0.004
	Negative PANSS	−3.31	0.09	0.226

#### Results of sensitivity analyses

To check the robustness of our results for the primary outcome, we performed an analysis on studies that were judged to have fair or good methodological and reporting quality. Most of these studies were in favor of decreased MNA in cases, although this finding was not statistically significant (13/18; *P* = 0.096).

### Publication bias

Given the multi-modal nature of the included studies, it was not possible to use statistical tests or funnel plots to check for the possible role of publication bias in our results. We figured the best way for checking any potential publication bias in our review is to check for the time-lag phenomenon, defined as “an initial wave of studies reporting positive or expected results, followed by a secondary wave of negative results” which is an indicator of possible publication bias (107). Our investigations on a quarter of the most recent included studies [8 studies (75, 96, 97, 100, 102–105), from 2018 to 2021] revealed that 87.5% (7/8 studies) were in the same direction as the results of our main analysis (reduced MNA, altered MNS). Although this finding does not rule out the publication bias for certain, it ascertains the absence of it to some considerable degree.

### Certainty of evidence

For the primary outcome, we believed there were some concerns for the “methodological limitations” domain as the considerable presence of bias across the included studies might have affected our results. We also believed there were minor concerns for the “coherence” domain because some studies reported contradictory results. No concerns were identified for the “adequacy” and “relevance” domains. Overall, given that the frequent presence of bias across the included studies might have affected our results, we decided to downgrade the certainty of the evidence by one level because of the “methodological limitations” domain. Thus, we believe there was moderate confidence in our findings.

## Discussion

### Interpretation of the results

#### MNA in schizophrenia; What did we find?

Mehta et al. (54) conducted a systematic review on the same subject in 2014. However, to our knowledge, this is the first systematic review that has also incorporated qualitative data analysis to evaluate the mirror mechanism in schizophrenia patients and identify some of the possible sources of heterogeneity in the findings.

Some hypothesize that schizophrenia might be a disorder of the “social brain” (108). Mirror neurons are collections of neurons that are believed to be part of this social brain network (109). From this point of view, it has been hypothesized that MNA is impaired in schizophrenia. Our study reveals, with moderate confidence, that there is indeed an impaired MNA system in these patients.

#### Contradictory results; Some possible explanations

Although most findings were in the same direction, one might question why others found contradictory results. We aim to describe here some of the potential causes underlying those results.

First, our meta-regression analyses found a statistically significant relationship between the mean positive PANSS scores and age of the participants in each study and the direction of the observed effect in that study. Studies that demonstrated intact/increased MNA enrolled patients with higher positive PANSS scores and age, compared to the studies that demonstrated decreased MNA.

Regarding the relationship between the positive PANSS scores and the direction of the effect, our results indicate that patients with higher positive PANSS scores are more likely to have intact/increased MNA. Positive PANSS measures the severity of the positive symptoms of the disorder, such as delusions, conceptual disorganization, hallucinations, and hostility (62). McCormick et al. (87) found a similar pattern in their study, suggesting that MNS may be overactive when positive symptoms are most prevalent (especially hallucinations). Mitra et al. (89) reported a negative correlation between the mu wave suppression and the thought disturbance cluster on PANSS, proposing that according to the theory that dopamine levels in the brain and the performance of the brain circuits have an “inverted-U” shaped relationship, an increase in brain dopamine levels during schizophrenia possibly disrupts the MNS circuit, leading to psychopathology manifestations. Other studies did not find a significant correlation between positive PANSS scores and MNA (74, 78, 80, 84, 86, 88, 90, 96, 99, 102). These contradictory results could be due to differences in experimental conditions, stage of disease, or the measures used to assess symptoms. Nevertheless, the idea of MNA correlating with patients' symptoms seems to be a plausible hypothesis. Indeed, this hypothesis was previously mentioned by Mehta et al. (54), making it an explanation worth further investigation. This is also in line with Frith and Corcoran's theoretical model of the relationship between social cognitive processes and psychotic symptoms (110).

Regarding age, similar results were found previously in autism, suggesting that individuals with autism may outgrow any mirror neuron deficit after a certain age (111, 112), although some other studies question these results (113, 114). One recent study indicates that in general, there might be some differences in the MNA between younger and older adults (115), where older adults showed Mu suppression in frontal and frontotemporal regions during a memory task, in contrast with young adults who showed Mu enhancement. Besides these, some studies have also shown that the social cognitive performance of schizophrenia patients may actually increase by age (116). Linke et al. (117) found a similar pattern in their study as well, but after including the patient's age at onset in their models, they concluded that this observed increase in social cognitive performance is not really due to the patients' age, but it is actually due to their later onset of the disease, as older patients are usually those with a later onset of psychosis as well. This is in line with previous studies that revealed age at the onset of the disease is negatively correlated with patients' cognitive performance (118, 119).

In the study of Horan et al. (82–84), they used a mask before group-level analyses, which might “bias against finding significant between-group differences,” as stated by the authors. In the study of Andrews et al. (73), they used a combination of TMS and EEG that may have reduced the quality of the EEG signals from some participants. Also, the baseline stimulus used to directly compare the EEG and TMS measures (blank screen) was not the same for the two measures. The studies of McCormick et al. (87) and Quintana (92) found increased MNA in patients. Quintana (91) study made a controversial decision by excluding BOLD signal changes during incorrect responses. The authors proposed that patients may have a compensatory increase in MNA while correctly performing the task. In the study of McCormick et al. (87), subgroup analyses showed only a subgroup of patients had greater mu suppression, the active psychosis subgroup. These findings indicate the need for more research on these subgroups of schizophrenia patients.

Most notably, we found the results of EEG studies to be very contradictory. Some possible explanations for such results have been previously mentioned in the study of Hobson and Bishop (120). First, they suggested that because the mu frequency band overlaps with the alpha frequency band (which is sensitive to attentional fluctuation), mu suppression could potentially be confounded by changes in attentional engagement. They also report that there is little consistency in how the specific baseline against which mu suppression is assessed should be defined. Finally, they examined mu suppression in 61 typical adults and reported that even in an optimal evaluation condition, 16–21% of participants showed no mu suppression to action observation task. Overall, they concluded that mu suppression can be used to index the human MNS, but the effect seems to be weak and unreliable, and it may also easily be confounded by alpha rhythm suppression. More interestingly, a recent study found that observation tasks may sometimes elicit mu rhythm enhancement rather than suppression (121). All these results question the reliability of the EEG paradigm for assessing MNA. Also, the validity of the TMS/EEG paradigm has been seriously questioned by another recent study (122). With all of those in mind, we still didn't consider these paradigms as invalid in our bias assessment process, as this domain required subjective judgments (where we tried to be conservative) and there are still some counter-arguments supporting the possible reliability of these paradigms.

#### MNA and negative symptoms in schizophrenia

Negative symptoms account for a substantial portion of the morbidity associated with schizophrenia (123). Empirical research has argued for an association between negative symptoms and anomalous MNA (124). We found the same association in some of the studies included in this review (85, 86, 95, 98). The study of Singh et al. (95) found lower mu wave suppression to positively correlate with negative PANSS scores, suggesting MNS may be underactive when negative symptoms predominate. However, the study of Brown et al. (76) found a statistically significant correlation between mu wave suppression and negative PANSS scores in the opposite direction of Singh's et al. Also, the study of Kato et al. (85) reported a negative correlation between the amplitudes of root-mean-square (RMS) of MEG responses and negative PANSS scores. Finally, Park et al. (91) reported the presence of a negative correlation between the functional deficits in MNS and negative PANSS scores. Although we didn't find any significant correlation between MNA and negative PANSS scores (*P* = 0.226), future studies should provide an in-depth assessment of the relationship between these two factors.

#### MNA and communication skills in schizophrenia

Deficits in communications skills have been previously documented in schizophrenia (125), but there has not been a comprehensive explanation for the etiology of this phenomenon up to this date. Indeed, MNS has been linked to developing communication skills v*ia* integrating auditory, visual, and motor stimulation (126). A study by Cantisani et al. reported a negative linear association between resting-state cerebral blood flow in the left inferior and middle frontal gyrus of schizophrenia patients with their communication skills, measured through the Social and Occupational Functioning Assessment Scale (SOFAS) (127). Our results indicate that across the literature, the inferior frontal gyrus (IFG) was the most investigated area for MNA in schizophrenia patients, where most studies indicated decreased MNA detection in this area. Putting these findings together, the disruption in MNA might be suggested as a possible explanation for communication skills in schizophrenia patients. Further studies are required to validate this hypothesis.

#### MNA and echopraxia in schizophrenia

Echopraxia is the pathological repetition by imitation of the movements of another person. In the context of schizophrenia, it has been mostly associated with the catatonic form (128). A previous speculative paper by Pridmore et al. suggested that pathologically handled MNS-generated representations, especially in IFG, might be involved in this dysfunction (129). Indeed, this was in line with the findings of the study of Zaytseva et al. (101) where the authors reported altered mu rhythm suppression in the right frontal and central brain regions in patients with catatonic schizophrenia. More studies on catatonic patients in the future are suggested to further evaluate the validity of this finding.

#### MNS and the “plasticity” hypothesis in schizophrenia

Previously, some have argued that MNS might have a plastic feature (130), meaning that after receiving treatment, disruptions in this system might at least partially resolve. However, a study by Mitra et al. (131) found that following 8 weeks of antipsychotic treatment, no significant changes took place in the MNA of patients. This is partly in line with our results that revealed there was no significant MNA difference between medicated and drug-free patients. These indicate that even though antipsychotic medications may improve cognitive deficits for some schizophrenia patients, they may not affect MNA significantly.

#### Another commonality between autism and schizophrenia

Our results indicate that MNA is altered in schizophrenia patients, similar to the individuals with autism. This finding contributes to the efforts of exploring the dimensions of mental disorders to integrate many levels of information to understand the nature of mental health and illness, such as efforts taking place in the projects of RdoC (132).

#### Rehabilitation through MNS-based training

Deriving clinical impact from such results could be an existing area of research. In a pilot study in 2020 (133), Hadoush et al. evaluated the effect of bilateral anodal transcranial direct current stimulation (tDCS) applied over the MNS of autism patients. They concluded that this intervention has a moderate therapeutic effect on children with autism in terms of their sociability, behavior, health, and even physical conditions. This pilot study reveals the potential of new rehabilitation methods through MNS-based training, which might benefit patients. It might be interesting to evaluate if similar results could be obtained for schizophrenia patients.

Another study that evaluated the effect of add-on yoga therapy on schizophrenia patients, revealed that MNA increased in the intervention group following 6 weeks of yoga therapy. They also found significant improvements in social cognition composite score (SCCS), negative symptoms (SANS), and positive symptoms (SAPS). One hypothesis is that the improvements in those clinical symptoms might have been achieved through the training of the MNS. Indeed, a previous study on yoga therapy for 2 years on 12 autism patients (6 in the interventional arm and 6 in the comparator arm) revealed improvements in imitation and other social skills of the participants (134). The authors hypothesized that guided imitation of therapist body positions might have stimulated MNA, resulting in an improved sense of self. Investigating the causal relationship between such findings might benefit future research.

### Limitation of evidence

All the included studies used indirect measures of MNA. It is known that intracranial electrodes give the most reliable evidence of MNA, but understandably, such procedures cannot be used for research on humans. Nevertheless, the indirect nature of the assessment tools used in the included studies, compared to the definitive direct sell recording techniques, should be considered as a limitation of the evidence. Also, a considerable proportion of the included studies had a sample size of < 34, which decreased the power of their statistical analysis. By the way, some studies did not use valid and comprehensive inclusion/exclusion criteria, which might increase the chance of confounding in their results. Finally, a proportion of the included studies did not report if controls were matched with cases for handedness.

### Limitations of review processes

We acknowledge several limitations in our study. Firstly, we used a weak statistical test for our analyses. Although it requires mentioning that considering the wide range of assessment paradigms we included in this review, more powerful statistical tests were not feasible. Secondly, we didn't assess the same outcome in other populations with almost the same pathology (i.e., schizoaffective disorder). Finally, some important information was not reported in the included studies. We tried to reach out to the authors to ask for that information but did not get any response. Nevertheless, we believe that possible none of these methodological limitations would significantly change the overall conclusions of this review.

Overall, we acknowledge that presenting a quantified summary for such a highly debated and controversial topic, given so few studies with vastly different modalities, would have its challenges and may require some methodological innovations. With that in mind, we still believe that our study managed to provide a clearer picture of the current state of knowledge on this subject, while also pointing to some of the existing biases and limitations in the literature.

### Implications

From our findings, one can claim, with moderate confidence, that MNA is altered in schizophrenia patients. This finding provides clues for a more in-depth understanding of the disorder and helps find a more comprehensive revision of the underlying pathophysiology of psychosis spectrum disorders. As more findings are being discovered that help to achieve a more in-depth understanding of psychiatric disorders, adjustments to our definitions for these illnesses seem necessary. Future researchers may evaluate the same deficits in patients with other disorders (e.g., bipolar disorder, depression, etc.) to come up with a better understanding of the common features across these disorders and facilitate the process of finding new semantic definitions for psychiatric illnesses.

We also urge future researchers on this subject to try to compensate for the existing biases and limitations in the literature. This may include conducting studies with larger sample sizes, using rigor eligibility criteria to minimize confounding effects, and utilizing valid paradigms to ensure the reliability of the results. Also, research on deriving potential clinical impact using MNS-based training methods could be an exciting topic for future investigations.

## Data availability statement

The original contributions presented in the study are included in Supplementary material. Further inquiries can be directed to the corresponding author.

## Author contributions

AV: conception and coordination of the review, designing the protocol, search, study selection, data extraction, methodological and reporting quality assessment, analysis of evidence, interpretation of the results, assessing the certainty of the evidence, and writing the review. MMb: data extraction, methodological, and reporting quality assessment. AR: data extraction, methodological, and reporting quality assessment. NH and AH: study selection. MS: data extraction. MMo: data extraction and assessing the certainty of the evidence. All authors contributed to the article and approved the submitted version.

## Conflict of interest

The authors declare that the research was conducted in the absence of any commercial or financial relationships that could be construed as a potential conflict of interest.

## Publisher's note

All claims expressed in this article are solely those of the authors and do not necessarily represent those of their affiliated organizations, or those of the publisher, the editors and the reviewers. Any product that may be evaluated in this article, or claim that may be made by its manufacturer, is not guaranteed or endorsed by the publisher.
