# Supplementary material for: The mirror mechanism in schizophrenia: A systematic review and qualitative meta-analysis
Source: Front Psychiatry. 2022 Sep 21;13:884828. doi: 10.3389/fpsyt.2022.884828 (PMC9532849; doi:10.3389/fpsyt.2022.884828)
Supplement: Supplementary file 1 [file Table_1.DOCX]

| **Study ID** | **Direction** | **N (SCZ/HC)** | **Mean age** | **Medicated** | **+ PANSS** | **- PANSS** | **Setting** | **Paradigm** | **Experimental Condition** |
| --- | --- | --- | --- | --- | --- | --- | --- | --- | --- |
| **Microstructural methods** | | | | | | | | | |
| Tseng 2015 | Altered | 32/32 | 32 | ✓ | - | - | Inpatient | DSI | Microstructural data |
| ElShahawi 2020 | Altered | 15/15 | 29 | ✓ | - | - | Mixed | DWI/DTI | Microstructural data |
| Saito 2018 | Altered | 16/16 | 21 | ✓ | - | - | Mixed | DWI/DTI | Microstructural data |
| **Functional methods** | | | | | | | | | |
| Brown 2016 | **◀︎▶︎** | 17/17 | 40 | ✓ | 19 (7) | 25 (8) | Inpatient | EEG | (a) Rest: inanimate motion, (b) Action-observation: observing video clips of two people sitting at a table, transferring coins from one bowl to the other bowls at the table. |
| Horan 2014-2 | **◀︎▶︎** | 32/26 | 46 | ✓ | - | - | Outpatient | EEG | (a) Rest: inanimate motion (two bouncing balls), (b) Action-observation: hand movements, people playing a throw and catch game by throwing a ball to themselves, to each other, and to and from the observer. |
| McCormick 2012 | **▲** | 16/16 | 37 | ✓ | 17 (12) | 16 (10) | Inpatient | EEG | (a) Rest: watching snow-fall, (b) Action-observation: bouncing balls and hand movements. |
| Mitra 2014 | **▼** | 15/15 | 29 | ✕ | - | - | Inpatient | EEG | (a) Rest: White screen, (b) Action-observation: video of handshakes, repeated at a rate of 1 per second. |
| Moehring 2015 | **▼** | 15/15 | 35 | ✓ | 16 (4) | 20 (5) | Outpatient | EEG | (a) Action-observation: observing a static image of gestures of a hand for the rock–paper–scissors game, (b) Action-execution: participants actively executed hand gestures when stimuli depicting rock, paper, or scissors were displayed. |
| Singh 2011 | **▼** | 20/12 | 21 | ✓ | 15 (15) | 17 (13) | Outpatient | EEG | (a) Rest: inanimate motion (two bouncing balls), (b) Action-observation: hand movements, point light display animation of a jumping human, people playing a game of throw and catch. |
| Zaytseva 2017 | **▼** | 11/32 | 23 | ✓ | - | - | - | EEG | Imaginary representation of one’s own walking on a familiar street (2 min) followed by the subjects’ self-reports. |
| Varcin 2010 | **▼** | 25/25 | 42 | ✓ | 15 (13) | 16 (10) | Outpatient | EMG | Watching facial expressions of happiness and anger displayed in 4 male and 4 female faces, while EMG was recorded from zygomaticus major and corrugator supercilii. |
| Das 2011 | **▼** | 20/19 | 34 | ✓ | 10 (3) | 18 (5) | Inpatient | fMRI | 16 blocks: 8 experimental in which two triangles mimicked human behavior (bluffing, persuading, surprising, and mocking), and 8 controls in which two triangles moved randomly. |
| Ferri 2014 | **▼** | 22/22 | 28 | ≈ | 14 (4) | 12 (5) | Outpatient | fMRI | 336 trials where subjects watched either ‘emotion action’, ‘emotion’, or ‘action’ stimuli and 32 imitation trials where subjects were given a request to imitate either the action or the emotion. |
| He 2021 | **◀︎▶︎** | 17/18 | 32 | ✓ | 26 (17) | 16 (13) | Inpatient | fMRI | Two runs of 182 trials each. Each run consisted of 3 stimuli: (a) observing videos of an actor making incomprehensible Russian sentences with gestures, (b) making comprehensible German sentences without any gestures, (c) making German sentences with accompanying gestures. |
| Horan 2014-1 | **◀︎▶︎** | 23/23 | 47 | ✓ | - | - | Outpatient | fMRI | Five runs of 6 blocks, each block consisted of 6 trials (3 fingers and 3 faces). The trials required subjects to either (a) observe: observe finger movements or a facial expression, (b) imitate: imitate the fingers movement or the facial expression, and (c) execute: make the movement or facial expression described by each word. Words included the following in a random order: Lift Index, Lift Middle, Happy, Sad, Angry, Afraid. |
| Horan 2016 | **◀︎▶︎** | 21/21 | 47 | ✓ | - | - | Outpatient | fMRI | Four runs of a mixed blocked/event-related paradigm. Each run consisted of two components: (a): (i) observing videos of patients receiving a painful sound stimulation treatment; (ii) listening to the painful sounds (to create ROIs). (b): manipulations of perspective-taking (imagine ‘Self’ vs ‘Other’ experiencing pain) and cognitive appraisal (treatment was ‘Effective’ vs ‘Not Effective’). |
| Lee 2014 | **▼** | 15/16 | 37 | ✓ | 10 (3) | 13 (3) | Outpatient | fMRI | 180-trials (0.5s of watching phase for each); (a) observation phase: subjects watched either facial or word stimuli, (b) expression phase: subjects actively expressed the emotions displayed, (c) returning phase: subjects returned to neutral facial expression after watching a neutral cue on the screen. |
| Park 2009 | **▼** | 15/16 | - | ✓ | 13 (2) | 17 (4) | Outpatient | fMRI | 24 blocks; each block consisted of perceiving, inferring, and selecting appropriate responses (30s, 20s, and 10s respectively) to ambiguous or certain emotional events narrated by a graphical avatar. The neutral certain condition was the control condition. |
| Quintana 2001 | **▲** | 8/8 | 33 | ✓ | - | - | Outpatient | fMRI | Four runs of block-design paradigms – each run consisted of 3 resting blocks scattered among 2 sets (colored circles or drawings of facial expressions) of 6 task trials, where the subject was required to match the cues. |
| Stegmayer 2018 | **▼** | 22/25 | 38 | ✓ | 18 (7) | 19 (5) | Mixed | fMRI | Two runs of event-related paradigm – each run consisted of 3 phases: (a) visual command phase (3s), (b) planning phase (3s): participants had to plan movements, (c) execution phase (3s): participants should’ve executed the gesture that was stated in the visual command phase. |
| Thakkar 2014 | **▼** | 16/16 | 39 | ≈ | 14 (10) | 23 (12) | Inpatient | fMRI | Four runs of 14 blocks – each block consisted of 3 trials (3 movement conditions in each). Subjects were required to either execute actions of pressing buttons while viewing these stimuli or observe (a) a hand pressing buttons, (b) an image of a hand and a button box, (c) inanimate marks. |
| Wordecha 2018 | **▼** | 25/26 | 35 | ✓ | 11 (3) | 18 (4) | Outpatient | fMRI | 112 trials – each trial consisted of (a) watching phase: watching animations displaying actions of agents presented as point-light walkers, (b) behavioral response phase: responding to the question ‘Are the two persons acting together or separately?’, (c) ISI phase. |
| Kato 2011 | **▼** | 15/15 | 33 | ✕ | 18 (4) | 18 (8) | - | MEG | (a) Rest: eyes fixed on a cross, (b) Action-observation: mouth opening movements. |
| Schurmann 2007 | **▼** | 11/11 | 54 | ✓ | - | - | Outpatient | MEG | (a) Rest: resting in a relaxed state, (b) Action-observation: manipulation of a small object with a hand; (c) Action-execution: participants manipulated the small object with their hand. |
| Andreasen 2008 | **▼** | 18/13 | 30 | ✕ | 12 (11) | 9 (8) | Outpatient | PET | Subjects were asked to say narrative stories explaining a given social situation. The control task required subjects to read aloud a neutral story that was presented on the monitor. |
| Choe 2018 | **▼** | 26/26 | 23 | ≈ | 16 (4) | 16 (4) | Outpatient | rs-fMRI | Resting-state |
| Gou 2014 | **▼** | 69/62 | 31 | ✓ | 12 (5) | 14 (6) | Inpatient | rs-fMRI | Resting-state |
| Park 2021 | **▼** | 37/80 | 23 | ≈ | 16 (4) | 17 (5) | Outpatient | rs-fMRI | Resting-state |
| Schilbach 2016 | **▼** | 116/133 | 34 | ✓ | - | - | Multi-centric | rs-fMRI | Resting-state |
| Sun 2021 | **▼** | 28/22 | 17 | ✕ | 23 (7) | 17 (7) | Inpatient | rs-fMRI | Resting-state |
| Bagewadi 2019 | **▼** | 30/28 | 27 | ✓ | 21 (16) | 20 (16) | Inpatient | TMS | (a) Rest: observing a static image, (b) Natural action-observation: a key held in pinch grasp, performing locking and unlocking, (c) Context-based action-observation: observing a video clip of a mother trying to unlock the door of a house that is on fire and her child is stuck in calling for help. |
| Enticott 2008 | **▼** | 15/15 | 38 | ✓ | 15 (4) | 15 (5) | - | TMS | (a) Rest: not specified, (b) Action-observation: non-goal directed and goal-directed finger movements. |
| Mehta 2014 | **▼** | 54/45 | 31 | ≈ | 24 (6) | 23 (9) | Mixed | TMS | (a) Rest: observing a static image, (b) Action-observation: a key held in pinch grasp, performing locking and unlocking movements. |
| Andrews 2015 | **◀︎▶︎** | 19/19 | 41.0 | ✓ | 16 (6) | 16 (5) | Outpatient | TMS/EEG | (a) Rest: observing a black screen, (b) Action-observation: 6 video clips: 2 static hands; a hand reaching out and clasping a mug; a hand pantomiming clasping a mug; and 2 interactive movements, one with hands from two different people, and a similar movement carried out by one person. |
