# Supplementary material for: The mirror mechanism in schizophrenia: A systematic review and qualitative meta-analysis
Source: Front Psychiatry. 2022 Sep 21;13:884828. doi: 10.3389/fpsyt.2022.884828 (PMC9532849; doi:10.3389/fpsyt.2022.884828)
Supplement: Supplementary file 2 [file Table_2.DOCX]

| **Study ID** | **Modality** | **PMv** | **IFG** | **IPL** | **STG** | **Insula** |
| --- | --- | --- | --- | --- | --- | --- |
| **Andreasen 2008** | PET | - | Lower | - | - | - |
| **Das 2011** | Task-based fMRI | - | Lower | Lower | Lower | - |
| **Ferri 2014** | Task-based fMRI | - | Lower | Lower | - | Lower |
| **Kato 2011** | MEG | - | - | Lower | - | - |
| **Lee 2014** | Task-based fMRI | Lower | Lower | Higher | - | Higher |
| **Park 2009** | Task-based fMRI | Lower | Lower | - | - | - |
| **Quintana 2001** | Task-based fMRI | Higher | Higher | - | - | - |
| **Schurmann 2007** | MEG | Lower | - | - | - | - |
| **Stegmayer 2018** | Task-based fMRI | - | Lower | Higher | - | - |
| **Thakkar 2014** | Task-based fMRI | - | - | Higher | Lower | - |
| **Wordecha 2018** | Task-based fMRI | - | - | - | Lower | - |
