# Supplementary material for: The mirror mechanism in schizophrenia: A systematic review and qualitative meta-analysis
Source: Front Psychiatry. 2022 Sep 21;13:884828. doi: 10.3389/fpsyt.2022.884828 (PMC9532849; doi:10.3389/fpsyt.2022.884828)
Supplement: Supplementary file 3 [file Table_3.DOCX]

| **Dependent variable** | **Independent variable** | **Β_0_ (Intercept)** | **β_1_** | **p-value** |
| --- | --- | --- | --- | --- |
| The direction of the effect | Age | -7.42 | 0.16 | <0.001 |
|  | Female to male ratio | -0.82 | -1.86 | 0.070 |
|  | Positive PANSS | -4.75 | 0.17 | 0.004 |
|  | Negative PANSS | -3.31 | 0.09 | 0.226 |
