# Supplementary material for: The mirror mechanism in schizophrenia: A systematic review and qualitative meta-analysis
Source: Front Psychiatry. 2022 Sep 21;13:884828. doi: 10.3389/fpsyt.2022.884828 (PMC9532849; doi:10.3389/fpsyt.2022.884828)
Supplement: Supplementary file 4 [file Data_Sheet_1.PDF]

Embase (via Ovid) <1947 to 08.12.2022>
1 (mirror neuron system).mp.
2 (mirror system).mp.
3 (motor resonance).mp.
4 (neuron adj5 mirror).mp.
5 (brain adj5 mirror).mp.
6 exp mirror neuron/
7 1 or 2 or 3 or 4 or 5 or 6
8 exp schizophrenia spectrum disorder/
9 (schizo*).mp.
10 (psychosis adj5 spectrum).mp.
11 8 or 9 or 10
12 7 and 11

PubMed <1996 to 08.12.2022>
(mirror neuron system[tw] OR Mirror Neurons[mh] OR mirror neuron*[tw] OR mirror system[tw] OR motor resonance[tw]) AND (schizo*[tw] OR Schizophrenia[mh] OR psychosis [tw])

Web of Science Core Collection <1900 to 08.12.2022>
#1. TS=”mirror neuron system”
#2. TS=”mirror system”
#3. TS=”motor resonance”
#4. TS=(neuron NEAR/5 mirror)
#5. TS=(brain NEAR/5 mirror)
#6. #1 OR #2 OR #3 OR #4 OR #5
#7. TS=(schizo*)
#8. TS=(psychosis NEAR/5 spectrum)
#9. #7 OR #8
#10. #6 AND #9
