# Supplementary material for: The mirror mechanism in schizophrenia: A systematic review and qualitative meta-analysis
Source: Front Psychiatry. 2022 Sep 21;13:884828. doi: 10.3389/fpsyt.2022.884828 (PMC9532849; doi:10.3389/fpsyt.2022.884828)
Supplement: Supplementary file 5 [file Data_Sheet_2.pdf]

Reviewers' arguments regarding paradigm validity and quality of report in each included study

In this file, we have presented a summary of the methods used in each included study to measure the mirror neuron activity and report our arguments for judging the validity of those methods. To check the full description of the methods used in each study, readers are encouraged to read the respective papers.

Andereasen 2008[1]

Class: Positron emission tomography (PET)
Methods: Quantitative PET blood flow data were acquired on a gradient-echo 4096-plus whole-body scanner following the bolus injection of [^{15}O] water. A sham study (patients were asked to read a list of words presented on the monitor) was performed to familiarize the participants with the procedures and reduce anxiety. Cerebral blood flow was calculated on a pixel-by-pixel basis using the autoradiographic method and normalized by dividing by global cerebral blood flow. An 18-mm Hanning filter was applied to reduce anatomical variability. The experimental task involved asking the participant to ''make up a story,'' using a situation that would induce them to attribute mental states to another person.
Reviewers' argument: Previously, it has been demonstrated that a PET scan, if conducted under standard protocols, is as precise as functional magnetic resonance imaging (fMRI) in measuring regional cerebral blood flow (rCBF)[2]. Furthermore, the task of constructing a hypothetical situation of projecting our mental states onto others is in line with the theory of mind (TOM) which has been associated with mirror neurons[3].
Reviewers' decision: The paradigm is valid.

Andrews 2015[4]

Class: Transcranial magnetic stimulation (TMS) + Electromyography (EMG) + Electroencephalography (EEG)
Methods: Participants were seated in front of a monitor placed at eye level. EEG electrodes were positioned over the sensorimotor cortex (C3, CZ, and C4) to measure the mu rhythm. Eye movement artifacts were detected using electrooculogram (EOG) electrodes. Ten TMS pulses were initially administered to the left primary motor cortex (M1) while participants were viewing a black screen, as a measure of general corticospinal excitability (CSE). Two blocks of videos, containing two static hands, a hand reaching out and clasping a mug, a hand pantomiming clasping a mug (without the mug), and two interactive movements (one with hands from two people, and a similar movement by one person), were shown to the participants with a short rest break in between to reduce fatigue. TMS was administered and EEG was recorded during the presentation of videos. The size of the TMS-induced motor-evoked potentials (MEPs) when observing an action was used as an index of mirror neuron activity. MEPs were recorded from the right abductor pollicis brevis (APB) muscle. To prevent the EEG trace from being affected by artifacts from the TMS, a sample and hold circuit that latched the EEG signal from before the TMS pulse and holds it at this level during the TMS pulse, was employed.
Reviewers' argument: TMS has very high temporal precision that allows a researcher to infer when activity in the pathways to muscles is modulated relative to action observation and also gives a close indication as to whether the responses found, correspond to the muscles involved in the observed action. Also, by measuring the amplitude of MEPs during action observation, one can infer whether activity in the muscle increases or decreases from baseline. Previous studies have found when single TMS pulses are applied to the primary motor cortex (M1), increases in MEPs in contralateral hand muscles are larger when individuals view hand actions compared to when TMS is applied at rest[5–7]. Therefore, these increases in MEP sizes during action observation have been regarded as an index of mirror neuron activity. On the other hand, mu rhythm suppression during action observation has been widely associated with mirror neuron activity[8–10]. Also, this study utilized some methods to ensure that the EEG trace was not affected by artifacts from the TMS.
Reviewers' decision: The paradigm is valid.

Bagewadi 2019[11]

Class: TMS + EMG
Methods: Subjects were made to sit on a chair in front of a laptop monitor. Single-pulse and paired-pulse TMS was delivered over the left primary motor cortex when subjects observed three experimental observation blocks: rest block (observing an image of the dorsal aspect of a hand and a lock), neutral action-observation block (observing a video depicting a hand holding a key in a lateral pinch to perform repeated locking/unlocking actions), and context-based action-observation block (observing three consecutive runs of a video depicting social and emotional stimuli and then goal-directed actions involving a person trying to unlock a door, with the key getting stuck in the keyhole. MEPs were recorded in the right first dorsal interosseus (FDI) muscle as an indicator of mirror neuron activity.
Reviewers' argument: As stated before, MEPs during action observation in contralateral hand muscles following TMS applied over the M1 cortex could be used as an indicator of mirror neuron activity.
Reviewers' decision: The paradigm is valid.

Brown 2016[12]

Class: EEG

Methods: Participants sat in front of a computer screen while they watched a series of videos depicting two people sitting facing each other at a table, transferring coins from one bowl to one of three other bowls in the middle of the table: one labeled with a "+", one with a "–", and one with a "0". Actions were seen both from a first-person or third-person point of view. Participants were told that each time a coin was put into the "+" bowl, they would receive a reward and when a coin was put into the "–" bowl, they would receive a punishment. When a coin was put into the "0" bowl, there would be no reward or punishment. EEG was recorded during the action observation task from the electrodes overlaying the sensorimotor cortex (C3, Cz, and C4). Mu rhythm suppression was measured as the index of mirror neuron activity.
Reviewers' argument: As stated before, mu rhythm suppression during action observation has been widely associated with mirror neuron activity.
Reviewers' decision: The paradigm is valid.

Choe 2018[13]

Class: Task-based functional magnetic resonance imaging (fMRI)
Methods: A Korean version of the Strange Story task[14], which is used to measure the theory of mind of the participant, was implemented. Functional MR images were acquired during the task. The regions of interest (ROIs) used for the fMRI analysis as the mirror neuron system network were obtained from a meta-analysis of neuroimaging studies on action observation and action imitation[15].
Reviewers' argument: Though the task seems relevant, acquisition parameters were not reported by the authors. As the acquisition parameters could have a substantial effect on the quality of the images obtained, it is not possible to evaluate the validity of the paradigm used in this study.
Reviewers' decision: The paradigm is considered invalid to the poor reporting of the parameters.

Das 2011[16]

Class: Task-based fMRI
Methods: Participants viewed blocks of silent animation using two triangles moving about the computer screen against a framed white background. Two types of animations were used: 4 TOM animations in which the two triangles mimicked human behavior (e.g., bluffing, persuading, surprising, and mocking one another), and 4 control animations in which the two triangles moved randomly. fMRI scan was conducted during the observation of the videos and task-specific regional responses were compared between the groups.
Reviewers' argument: The task was previously used in other studies to evaluate TOM[17–19], which has been proposed to be associated with mirror neuron activity.
Reviewers' decision: The paradigm is valid.

ElShahawi 2020[20]

Class: Diffusion-weighted imaging/Diffusion tensor imaging (DWI/DTI)
Methods: The pattern of diffusivity was compared between the groups in three ROIs: anterior cingulate gyrus (ACC), inferior parietal lobe (IPL), and premotor area (PMA).
Reviewers' argument: All these ROIs are believed to be parts of the mirror neuron system.
Reviewers' decision: The paradigm is valid.

Enticott 2008[21]

Class: TMS + EMG
Methods: Participants were positioned in front of a monitor. They were first administered 14 pulses of TMS to the left M1 cortex while at rest (baseline MEP). Then, single-pulse TMS was administered during the presentation of video clips that showed right-hand APB activity (lateral thumb movement to and from index finger, pen grasp, and hand-writing), and MEPs were recorded from the APB muscle.
Reviewers' argument: The methods used in this study are similar to those of Andrews 2015, although no EEG recording was conducted in this study.
Reviewers' decision: The paradigm is valid.

Ferri 2014[22]

Class: Task-based fMRI
Methods: The experimental stimuli consisted of 3 sets of videos: (1) 'emotion action', showing an actor) grasping an object (bottle, pencil case, receiver, or CD case) with the right hand and facially expressing anger, happiness, or no emotion; (2) 'emotion', showing only the face of the actor expressing anger, happiness, or no emotion, and (3) 'action', showing only the hand action. fMRI scans were conducted when the subjects were observing the videos. Neural activity in the following areas was compared between the groups: bilateral middle frontal gyrus, the right IFG (parts orbitalis, opercularis, and tri-angularis), the left PCG, and the left inferior parietal lobe.
Reviewers' argument: The task involves action observation which involves mirror neuron activity and the brain regions assessed are associated with the mirror neuron system.
Reviewers' decision: The paradigm is valid.

Guo 2014[23]

Class: Resting-state fMRI (rs-fMRI)
Methods: Subjects underwent functional MRI scans in the resting state, and brain-wide functional connectivity (FC) patterns, especially in the mirror neuron system ROIs, were compared between the groups.
Reviewers' argument: Previous studies have found that FC analysis using rs-fMRI scans may be used as a valid method for assessing the pattern of connectivity in the mirror neuron system[24–26].
Reviewers' decision: The paradigm is valid.

Horan 2014-1[27]

Class: Task-based fMRI
Methods: Two tasks were used: Finger tasks: (1) Observe the movement of fingers in a set of videos, (2) Imitating the movement of each finger with the right hand, and (3) make the movements described by words on the screen ("Lift Index" and "Lift Middle") with the right hand. Face tasks: (1) Observing the expressions on faces in a set of pictures, (2) Imitating the expression of those faces, and (3) making the expression described by words on the screen ("Happy", "Sad", "Angry", and "Afraid"). fMRI scans were obtained during the tasks and patterns of activity in mirror neuron system ROIs were compared between the groups.
Reviewers' argument: The tasks used in this study include action observation, imitation, and execution which are all associated with mirror neuron activity. fMRI is also a valid tool for assessing the patterns of activity in brain regions.
Reviewers' decision: The paradigm is valid.

He 2021[28]

Class: rs-fMRI
Methods: Subjects underwent functional MRI scans in the resting state, and brain-wide FC patterns were compared between the groups in a whole-brain analysis.
Reviewers' argument: The methods used in this study are similar to those used in the study by Guo 2014.
Reviewers' decision: The paradigm is valid.

Horan 2014-2[29]

Class: EEG
Methods: Participants were sat against a monitor and six experimental conditions were performed. These included: (1) watching a moving hand, (2) imitating the moving hand, (3) watching a video depicting a social noninteracting situation, (4) watching a video depicting a social spectator, (5) watching a video depicting a social interacting situation, and (6) a baseline condition video of two bouncing balls. Mu suppression ratios were calculated for central (C3, Cz, and C4) sites over the sensorimotor cortex.
Reviewers' argument: As stated before, mu rhythm suppression during action observation has been widely associated with mirror neuron activity.
Reviewers' decision: The paradigm is valid.

Horan 2016[30]

Class: Task-based fMRI
Methods: Aversive tones of hypothetical patients under pain were played to the participants. Then, participants were shown a set of video clips. These clips (without sound) showed the faces listening to the sounds. Each video began with the individual displaying a neutral face that transitioned to a facial expression of strong pain in reaction to the sounds. Participants were instructed to watch the video clips and either imagine how they would feel if they were under that pain or imagine how the patients in the clips were feeling under the pain. fMRI scans were obtained and patterns of activity in ROIs were compared between the groups.
Reviewers' argument: This task has been used in at least one previous study[31], and is believed to activate the empathy mechanism in the participants, which has been associated with mirror neuron activity[32]. fMRI is also a valid tool for assessing the patterns of activity in brain regions.
Reviewers' decision: The paradigm is valid.

Kato 2011[33]

Class: Magnetoencephalography (MEG)
Methods: Participants observed a set of video clips that showed line-symmetrical mouth opening movements of another individual (action observation) with some rest blocks in between. During the presentation of the task, event-related magnetic signals were collected and compared between the groups.
Reviewers' argument: MEG data is known to have a high spatial and temporal resolution and could assess high-gamma activation modulation during action observation, which has been proposed as an index of mirror neuron activity[34].
Reviewers' decision: The paradigm is valid.

Lee 2014[35]

Class: Task-based fMRI
Methods: A trial contained 3 different phases: "watching," "expressing," and "returning". In the watching phase, face stimuli of either happiness, sadness, or meaningless expression or word stimuli of either "happiness," "sadness," or "eye mouth" (meaningless expression) were presented on a screen. During the expressing phase,

participants were asked to imitate the facial stimuli or make a facial expression as the word stimuli indicated. In the returning phase, a neutral cartoon face was presented to stop participants from making an expression. fMRI scans were performed during the tasks and the patterns of activity were compared between the groups.
Reviewers' argument: The task used in this study is purely about facial mimicry and not action observation. Although facial mimicry as an imitation task has been proposed to be associated with mirror neuron activity, some contradictory findings are suggesting that at least some forms of mimicry do not activate the mirror neuron system effectively (e.g., sad expressions do not activate the mirror neuron system)[36].
Reviewers' decision: We cannot ensure the validity of the paradigm.

McCormick 2012[37]

Class: EEG
Methods: Participants were sat against a monitor and were instructed to (1) observe a video of visual white noise, (2) observe a video of two bouncing balls, (3) observe a video of a person moving his right hand, (4) observe the movement of their right hand, and (5) observe a "live" person moving his right hand. During the task, EEG data was recorded from electrodes placed over the sensorimotor cortex (F3-C3 and F4-C4) and mu rhythm suppression patterns were compared between the groups.
Reviewers' argument: As stated before, mu rhythm suppression during action observation has been widely associated with mirror neuron activity.
Reviewers' decision: The paradigm is valid.

Mehta 2014[38]

Class: TMS + EMG
Methods: Participants sat in front of a monitor. Single-pulse and paired-pulse TMS were administered during the actual observation of action with active FDI, virtual observation of the same act, and rest state. MEPs were recorded from the APB muscle.
Reviewers' argument: The methods used in this study are similar to those of Enticott 2008.
Reviewers' decision: The paradigm is valid.

Mitra 2014[39]

Class: EEG
Methods: Participants sat against a monitor and watched a video clip containing white background at the beginning, two socially relevant biological motions, white visual noise, and a final sequence of white backgrounds. During the task, EEG data was recorded from electrodes placed over the sensorimotor cortex, and mu rhythm suppression patterns were compared between the groups.
Reviewers' argument: As stated before, mu rhythm suppression during action observation has been widely associated with mirror neuron activity.
Reviewers' decision: The paradigm is valid.

Moehring 2015[40]

Class: EEG + Event-related potentials (ERP)
Methods: Participants were asked to take a seat in a comfortable chair in front of the screen and observed static images of a hand forming gestures of the rock–paper–scissors game and to actively imitate hand gestures as soon as imperative stimuli depicting rock, paper, or scissors were displayed. During the task, EEG data were recorded from a 64-channel DC amplifier. Data were segmented for each experimental condition and averages were constructed for each experimental condition.
Reviewers' argument: A similar study design was previously reported and validated as a method for measuring mirror neuron activity within the human brain[41].
Reviewers' decision: The paradigm is valid.

Okruszek 2018[42]

Class: Task-based fMRI
Methods: Stimuli consisted of animations depicting the actions of point-light walkers (PLW). Half of the stimuli presented social interactions between agents (including the conventional use of communicative gestures, emotional situations, or synchronous activity of the agents during the games/dancing). The other half presented individual conditions such as physical movements (e.g., jumps, squats down) without impacting one another. Then, participants were asked if the two persons acted together or separately and responded by pressing the button of the response pad. fMRI scans were obtained and patterns of activity in ROIs associated with the mirror neuron system were compared between the groups.
Reviewers' argument: The task used in this study involves direct action observation and recognition of communicative interactions which are associated with mirror neuron activity[43].
Reviewers' decision: The paradigm is valid.

Park 2021[44]

Class: Task-based fMRI
Methods: TOM was measured with the short form of the false belief task. Each stage of the task included a picture with a short vignette, followed by comprehension and justification questions which measured the subject's ability to infer the character's mental state. The first-order task

evaluated the ability of the subject to recognize a character's false belief about reality and the second-order task assessed a character's knowledge of the mental state of the other character.
Reviewers' argument: This task evaluates the theory of mind (TOM) which has been associated with mirror neurons[3].
Reviewers' decision: The paradigm is valid.

Park 2009[45]

Class: Task-based fMRI
Methods: Each block consisted of perception, inference, and answer phases. In the perception phase, an avatar narrated a previous emotional event that caused the current emotional state with congruent emotionality in the emotional condition, and a previous neutral event without emotionality in the neutral condition. In the inference phase, participants were instructed to infer what event had previously happened to the avatar and then infer the previous event that caused the avatar's emotional or neutral behavior. In the answer phase, they were presented with sentences and responded with "true" or "false" according to whether or not the sentence matched the previous event. fMRI scans were performed during the task and patterns of activity in the ROIs associated with the mirror neuron system were compared between the groups.
Reviewers' argument: This task involved attributional style by having the participants imagine and reenact avatars' action representations, represent and process the avatars' sequential previous events, and deductively infer the situational causes. Attributional style has been associated with TOM skills in previous studies[46–48], which on its own has been associated with mirror neuron system activity.
Reviewers' decision: The paradigm is valid.

Quintana 2001[49]

Class: Task-based fMRI
Methods: Participants performed a simple visuomotor task where a visual cue (a color circle or a diagram of a facial expression) indicated the correct choice between two color circles or two facial diagrams presented a few seconds later. They responded via button-pressing (left or right) on a mouse device. fMRI scans were performed during the task and the patterns of activity were compared between the groups.
Reviewers' argument: This task design has been previously used for understanding the interactions between separate brain regions of distributed networks in monkeys[50,51]. The authors hypothesized that because of the relationship between facial affect recognition and social learning and adaptation, and given the known deficits in the latter in schizophrenia patients, compensatory mechanisms of increased neural activity in the mirror neuron system should be observed.
Reviewers' decision: We cannot ensure the validity of the paradigm.

Saito 2018[52]

Class: DWI/DTI
Methods: Fractional anisotropy and Trace of the tracts interconnecting three ROIs were compared between the groups: caudal middle frontal area, anterior cingulate gyrus (ACC), and inferior parietal lobe (IPL).
Reviewers' argument: All these ROIs are believed to be parts of the mirror neuron system.
Reviewers' decision: The paradigm is valid.

Schilbach 2016[53]

Class: rs-fMRI
Methods: Subjects underwent functional MRI scans in the resting state, and brain-wide FC patterns, especially in the mirror neuron system ROIs, were compared between the groups.
Reviewers' argument: The methods used in this study are similar to those used in the study by Guo 2014.
Reviewers' decision: The paradigm is valid.

Schurmann 2007[54]

Class: MEG
Methods: Neuromagnetic data were acquired during 3 experimental conditions: (1) rest, (2) the participants observed the experimenter manipulate a small object with her right hand, and (3) the participants manipulated the small object with their right hand without seeing their hand.
Reviewers' argument: Same as the Kato 2011 study.
Reviewers' decision: The paradigm is valid.

Singh 2011[55]

Class: EEG
Methods: Participants sat against a monitor and watched the following video clips: (1) baseline/ball condition, (2) moving hand condition, (3) social interactive condition, and (4) biological motion/point light display animation. During the task, EEG data was recorded from electrodes placed over the sensorimotor cortex (C3, Cz, C4, O1, and O2), and mu rhythm suppression patterns were compared between the groups.
Reviewers' argument: As stated before, mu rhythm suppression during action observation has been widely associated with mirror neuron activity.

Reviewers' decision: The paradigm is valid.

Stegmayer 2018[56]

Class: Task-based fMRI
Methods: Participants were asked to perform some novel and some familiar gestures with their right hand when instructions were presented visually as written commands. fMRI scans were obtained and patterns of activity in ROIs were compared between the groups.
Reviewers' argument: The task was a pantomime gesture task. This study was built upon the hypothesis that alterations in the mirror neuron system may lead to poor gesture performance, as suggested by some previous studies[57–59]. fMRI is also a valid tool for assessing the patterns of activity in brain regions.
Reviewers' decision: The paradigm is valid.

Sun 2021[60]

Class: rs-fMRI
Methods: Subjects underwent functional MRI scans in the resting state, and dynamic FC (dFC) patterns, especially in the mirror neuron system ROIs, were compared between the groups.
Reviewers' argument: The methods used in this study are similar to those used in the study by Guo 2014, with the only difference being that in this study, the investigators used dFC instead of FC.
Reviewers' decision: The paradigm is valid.

Thakkar 2014[61]

Class: Task-based fMRI
Methods: Participants were instructed to watch a video of either a moving hand or a spatial cue and then to either execute finger movements associated with the stimulus or simply observe. fMRI scans were obtained and patterns of activity in ROIs associated with the mirror neuron system were compared between the groups.
Reviewers' argument: The task used in this study involves direct action observation and execution which are widely associated with mirror neuron activity.
Reviewers' decision: The paradigm is valid.

Tseng 2015[62]

Class: Diffusion spectrum imaging (DSI)
Methods: The cortical thickness and fiber tract integrity of the brain regions associated with the mirror neuron system were compared between the groups.
Reviewers' argument: -
Reviewers' decision: The paradigm is valid.

Varcin 2010[63]

Class: EMG
Methods: Surface EMG was used to record changes in the muscle activity over the left corrugator and zygomaticus regions while participants were seeing pictures of happy and angry facial expressions.
Reviewers' argument: Authors argued that the EMG data from these muscles could be used as an indirect measure of mirror neuron activity.
Reviewers' decision: We cannot ensure the validity of the paradigm.

Zaytseva 2017[64]

Class: EEG
Methods: The experimental task included an imaginary representation of one walking on a familiar street. It was followed by the subjects' self-reports. Mu rhythm suppression in the frontal sites (F3 and F4) and the sensorimotor cortex (C3 and C4) were compared between the groups.
Reviewers' argument: As stated before, mu rhythm suppression during action observation has been widely associated with mirror neuron activity. However, the details of the task used in this study, the acquisition parameters, and the analytical methods used were reported insufficiently.
Reviewers' decision: We cannot ensure the validity of the paradigm.

References

1. Andereasen, N., Calage, C. & O'Leary, D. Theory of Mind and Schizophrenia: A Positron Emission Tomography Study of Medication-Free Patients. *Schizophr Bull* **35**, 1030–1030 (2009).
2. Feng, C.-M. *et al.* CBF changes during brain activation: fMRI vs. PET. *Neuroimage* **22**, 443–446 (2004).
3. Acharya, S. & Shukla, S. Mirror neurons: Enigma of the metaphysical modular brain. *J Nat Sci Biol Med* **3**, 118 (2012).
4. Andrews, S. C., Enticott, P. G., Hoy, K. E., Thomson, R. H. & Fitzgerald, P. B. No evidence for mirror system dysfunction in schizophrenia from a multimodal TMS/EEG study. *Psychiatry Res* **228**, 431–440 (2015).
5. Strafella, A. P. & Paus, T. Modulation of cortical excitability during action observation. *Neuroreport* **11**, 2289–2292 (2000).
6. Patuzzo, S., Fiaschi, A. & Manganotti, P. Modulation of motor cortex excitability in the left hemisphere during action observation: a single- and paired-pulse transcranial magnetic stimulation study of self- and non-self-action observation. *Neuropsychologia* **41**, 1272–1278 (2003).
7. Fadiga, L., Craighero, L. & Olivier, E. Human motor cortex excitability during the perception of others' action. *Curr Opin Neurobiol* **15**, 213–218 (2005).
8. Muthukumaraswamy, S. D., Johnson, B. W. & McNair, N. A. Mu rhythm modulation during observation of an object-directed grasp. *Cognitive Brain Research* **19**, 195–201 (2004).
9. Muthukumaraswamy, S. D. & Johnson, B. W. Changes in rolandic mu rhythm during observation of a precision grip. *Psychophysiology* **41**, 152–156 (2004).
10. Kladi, A., Iliadou, P., Tsolaki, M. & Moraitou, D. Age-related Differences in Mu Rhythm During Emotional Destination Memory Task. *Curr Aging Sci* **15**, 26–36 (2022).
11. Bagewadi, V. I. *et al.* Diminished modulation of motor cortical reactivity during context-based action observation in schizophrenia. *Schizophr Res* **204**, 222–229 (2019).
12. Brown, E. C., Gonzalez-Liencres, C., Tas, C. & Brüne, M. Reward modulates the mirror neuron system in schizophrenia: A study into the mu rhythm suppression, empathy, and mental state attribution. *Soc Neurosci* **11**, 175–186 (2016).
13. Choe, E. *et al.* Aberrant within- and between-network connectivity of the mirror neuron system network and the mentalizing network in first episode psychosis. *Schizophr Res* **199**, 243–249 (2018).
14. Happé, F. G. E. An advanced test of theory of mind: Understanding of story characters' thoughts and feelings by able autistic, mentally handicapped, and normal children and adults. *J Autism Dev Disord* **24**, 129–154 (1994).
15. Caspers, S., Zilles, K., Laird, A. R. & Eickhoff, S. B. ALE meta-analysis of action observation and imitation in the human brain. *Neuroimage* **50**, 1148–1167 (2010).
16. Das, P., Lagopoulos, J., Coulston, C. M., Henderson, A. F. & Malhi, G. S. Mentalizing impairment in schizophrenia: A functional MRI study. *Schizophr Res* **134**, 158–164 (2012).
17. Castelli, F. Autism, Asperger syndrome and brain mechanisms for the attribution of mental states to animated shapes. *Brain* **125**, 1839–1849 (2002).

18. Malhi, G. S. *et al.* A functional MRI study of Theory of Mind in euthymic bipolar disorder patients. *Bipolar Disord* **10**, 943–956 (2008).
19. Livingston, L. A., Shah, P., White, S. J. & Happé, F. Further developing the Frith–Happé animations: A quicker, more objective, and web-based test of theory of mind for autistic and neurotypical adults. *Autism Research* **14**, 1905–1912 (2021).
20. ElShahawi, H. H., Sakr, H. M., Hashim, M. A., Mohamed, H. H. & Abdeen, M. S. Social cognition correlation to white matter integrity alteration in mirror neurons of schizophrenic patients: DTI study. *Neurol Psychiatry Brain Res* **38**, 65–73 (2020).
21. ENTICOTT, P. *et al.* Reduced motor facilitation during action observation in schizophrenia: A mirror neuron deficit? *Schizophr Res* **102**, 116–121 (2008).
22. Ferri, F. *et al.* Binding Action and Emotion in First-Episode Schizophrenia. *Psychopathology* **47**, 394–407 (2014).
23. Guo, S., Kendrick, K. M., Yu, R., Wang, H.-L. S. & Feng, J. Key functional circuitry altered in schizophrenia involves parietal regions associated with sense of self. *Hum Brain Mapp* **35**, 123–139 (2014).
24. Plata-Bello, J. *et al.* The mirror neuron system also rests. *Brain Struct Funct* **222**, 2193–2202 (2017).
25. McGregor, H. R. & Gribble, P. L. Changes in visual and sensory-motor resting-state functional connectivity support motor learning by observing. *J Neurophysiol* **114**, 677–688 (2015).
26. Dai, J., Li, C. & Zhai, H. Development of the functional connectivity of the frontoparietal mirror neuron network in preschool Children: An investigation under resting state. *Journal of Clinical Neuroscience* **70**, 214–220 (2019).
27. Horan, W. P. *et al.* Self-reported empathy and neural activity during action imitation and observation in schizophrenia. *Neuroimage Clin* **5**, 100–108 (2014).
28. He, Y. *et al.* Modality-specific dysfunctional neural processing of social-abstract and non-social-concrete information in schizophrenia. *Neuroimage Clin* **29**, 102568 (2021).
29. Horan, W. P., Pineda, J. A., Wynn, J. K., Iacoboni, M. & Green, M. F. Some markers of mirroring appear intact in schizophrenia: evidence from mu suppression. *Cogn Affect Behav Neurosci* **14**, 1049–1060 (2014).
30. Horan, W. P. *et al.* Pain empathy in schizophrenia: an fMRI study. *Soc Cogn Affect Neurosci* **11**, 783–792 (2016).
31. Lamm, C., Batson, C. D. & Decety, J. The Neural Substrate of Human Empathy: Effects of Perspective-taking and Cognitive Appraisal. *J Cogn Neurosci* **19**, 42–58 (2007).
32. Bekkali, S. *et al.* Is the Putative Mirror Neuron System Associated with Empathy? A Systematic Review and Meta-Analysis. *Neuropsychol Rev* **31**, 14–57 (2021).
33. Kato, Y. *et al.* Magnetoencephalography Study of Right Parietal Lobe Dysfunction of the Evoked Mirror Neuron System in Antipsychotic-Free Schizophrenia. *PLoS One* **6**, e28087 (2011).
34. Dreyer, A. M. & Rieger, J. W. High-gamma mirror activity patterns in the human brain during reach-to-grasp movement observation, retention, and execution—An MEG study. *PLoS One* **16**, e0260304 (2021).
35. Lee, J. S., Chun, J. W., Yoon, S. Y., Park, H.-J. & Kim, J.-J. Involvement of the mirror neuron system in blunted affect in schizophrenia. *Schizophr Res* **152**, 268–274 (2014).

36. Likowski, K. U. *et al.* Facial mimicry and the mirror neuron system: simultaneous acquisition of facial electromyography and functional magnetic resonance imaging. *Front Hum Neurosci* **6**, (2012).
37. McCormick, L. M. *et al.* Mirror neuron function, psychosis, and empathy in schizophrenia. *Psychiatry Res Neuroimaging* **201**, 233–239 (2012).
38. Mehta, U. M., Thirthalli, J., Basavaraju, R., Gangadhar, B. N. & Pascual-Leone, A. Reduced Mirror Neuron Activity in Schizophrenia and Its Association With Theory of Mind Deficits: Evidence From a Transcranial Magnetic Stimulation Study. *Schizophr Bull* **40**, 1083–1094 (2014).
39. Mitra, S., Nizamie, S. H., Goyal, N. & Tikka, S. K. Mu-wave Activity in Schizophrenia: Evidence of a Dysfunctional Mirror Neuron System from an Indian Study. *Indian J Psychol Med* **36**, 276–281 (2014).
40. Möhring, N. *et al.* Mirror neuron deficit in schizophrenia: Evidence from repetition suppression. *Schizophr Res* **168**, 174–179 (2015).
41. Dinstein, I., Hasson, U., Rubin, N. & Heeger, D. J. Brain Areas Selective for Both Observed and Executed Movements. *J Neurophysiol* **98**, 1415–1427 (2007).
42. Okruszek, Ł. *et al.* Brain correlates of recognition of communicative interactions from biological motion in schizophrenia. *Psychol Med* **48**, 1862–1871 (2018).
43. Centelles, L., Assaiante, C., Nazarian, B., Anton, J.-L. & Schmitz, C. Recruitment of Both the Mirror and the Mentalizing Networks When Observing Social Interactions Depicted by Point-Lights: A Neuroimaging Study. *PLoS One* **6**, e15749 (2011).
44. Park, S. H. *et al.* Intrinsic cerebellar functional connectivity of social cognition and theory of mind in first-episode psychosis patients. *NPJ Schizophr* **7**, 59 (2021).
45. Park, K.-M. *et al.* Neural basis of attributional style in schizophrenia. *Neurosci Lett* **459**, 35–40 (2009).
46. Berry, K., Bucci, S., Kinderman, P., Emsley, R. & Corcoran, R. An investigation of attributional style, theory of mind and executive functioning in acute paranoia and remission. *Psychiatry Res* **226**, 84–90 (2015).
47. Boyden, P., Knowles, R., Corcoran, R., Hamilton, S. & Rowse, G. A preliminary investigation into theory of mind and attributional style in adults with grandiose delusions. *Cogn Neuropsychiatry* **20**, 109–121 (2015).
48. Jeon, I. H. *et al.* Attributional Style in Healthy Persons: Its Association with 'Theory of Mind' Skills. *Psychiatry Investig* **10**, 34 (2013).
49. Quintana, J. A Compensatory Mirror Cortical Mechanism for Facial Affect Processing in Schizophrenia. *Neuropsychopharmacology* **25**, 915–924 (2001).
50. Quintana, J. & Fuster, J. M. Mnemonic and predictive functions of cortical neurons in a memory task. *Neuroreport* **3**, 721–724 (1992).
51. Quintana, J. & Fuster, J. M. Spatial and Temporal Factors in the Role of Prefrontal and Parietal Cortex in Visuomotor Integration. *Cerebral Cortex* **3**, 122–132 (1993).
52. Saito, Y. *et al.* Impaired white matter connectivity between regions containing mirror neurons, and relationship to negative symptoms and social cognition, in patients with first-episode schizophrenia. *Brain Imaging Behav* **12**, 229–237 (2018).
53. Schilbach, L. *et al.* Differential Patterns of Dysconnectivity in Mirror Neuron and Mentalizing Networks in Schizophrenia. *Schizophr Bull* **42**, 1135–1148 (2016).

54. Schürmann, M. *et al.* Manifest disease and motor cortex reactivity in twins discordant for schizophrenia. *British Journal of Psychiatry* **191**, 178–179 (2007).
55. Singh, F., Pineda, J. & Cadenhead, K. S. Association of impaired EEG mu wave suppression, negative symptoms and social functioning in biological motion processing in first episode of psychosis. *Schizophr Res* **130**, 182–186 (2011).
56. Stegmayer, K. *et al.* Limbic Interference During Social Action Planning in Schizophrenia. *Schizophr Bull* **44**, 359–368 (2018).
57. Cabrera, M. E., Novak, K., Foti, D., Voyles, R. & Wachs, J. P. Electrophysiological indicators of gesture perception. *Exp Brain Res* **238**, 537–550 (2020).
58. Matthews, N., Gold, B. J., Sekuler, R. & Park, S. Gesture Imitation in Schizophrenia. *Schizophr Bull* **39**, 94–101 (2013).
59. Park, S., Matthews, N. & Gibson, C. Imitation, Simulation, and Schizophrenia. *Schizophr Bull* **34**, 698–707 (2007).
60. Sun, F. *et al.* Abnormal dynamic functional network connectivity of the mirror neuron system network and the mentalizing network in patients with adolescent-onset, first-episode, drug-naïve schizophrenia. *Neurosci Res* **162**, 63–70 (2021).
61. Thakkar, K. N., Peterman, J. S. & Park, S. Altered Brain Activation During Action Imitation and Observation in Schizophrenia: A Translational Approach to Investigating Social Dysfunction in Schizophrenia. *American Journal of Psychiatry* **171**, 539–548 (2014).
62. Tseng, C.-E. J. *et al.* Altered cortical structures and tract integrity of the mirror neuron system in association with symptoms of schizophrenia. *Psychiatry Res Neuroimaging* **231**, 286–291 (2015).
63. VARCIN, K. J., BAILEY, P. E. & HENRY, J. D. Empathic deficits in schizophrenia: The potential role of rapid facial mimicry. *Journal of the International Neuropsychological Society* **16**, 621–629 (2010).
64. Zaytseva, Y., Morozova, A., Bendova, M. & Garakh, Z. Is motor imagery different in catatonic schizophrenia? *Psych J* **6**, 137–138 (2017).
