# Supplementary material for: The mirror mechanism in schizophrenia: A systematic review and qualitative meta-analysis
Source: Front Psychiatry. 2022 Sep 21;13:884828. doi: 10.3389/fpsyt.2022.884828 (PMC9532849; doi:10.3389/fpsyt.2022.884828)
Supplement: Supplementary file 6 [file Data_Sheet_3.pdf]

Table of Contents

LIST OF ABBREVIATIONS 2

ANDEREASEN 2008 3
ANDREWS 2015 4
BAGEWADI 2019 5
BROWN 2016 6
CHOE 2018 7
DAS 2011 8
ELSHAHAWI 2020 9
ENTICOTT 2008 10
FERRI 2014 11
GUO 2014 12
HE 2021 13
HORAN 2014-1 14
HORAN 2014-2 15
HORAN 2016 16
KATO 2011 17
LEE 2014 18
MCCORMICK 2012 19
MEHTA 2014 20
MITRA 2014 21
MOEHRING 2015 22
OKRUSZEK 2018 23
PARK 2009 24
PARK 2021 25
QUINTANA 2001 26
SAITO 2018 27
SCHILBACH 2016 28
SCHURMANN 2007 29
SINGH 2011 30
STEGMAYER 2018 31
SUN 2021 32
THAKKAR 2014 33
TSENG 2015 34
VARCIN 2010 35
ZAYTSEVA 2017 36

REFERENCES 37

List of abbreviations

Abbreviation	Meaning
DSI	Diffusion spectrum imaging
DSM	Diagnostic and statistical manual of mental disorders
DTI	Diffusion tensor imaging
DWI	Diffusion weighted imaging
EEG	Electroencephalography
EMG	Electromyography
EOG	Electrooculogram
EPI	Echo-planar imaging
FA	Flip angle
fMRI	Functional magnetic resonance imaging
GE	Gradient-echo
HC	Healthy controls
ICD	International classification of diseases
IQ	Intelligence quotient
MEG	Magnetoencephalography
MEP	Motor evoked potential
MNS	Mirror neuron system
PET	Positron emission tomography
RMT	Resting motor threshold
rs-fMRI	Resting-state functional magnetic resonance imaging
SCZ	Schizophrenia
SE	Spin-echo
SI1mV	Stimulus intensity that is needed to evoke MEP of 1 mV
SMF	Static magnetic field
SR	Sampling rate
T-fMRI	Task-based functional magnetic resonance imaging
TE	Echo time
TMS	Transcranial magnetic stimulation
TR	Repetition time

Andereasen 2008 (1)

**Methods**	**Setting:** Mental health clinical research center at the University of Iowa<br>**Country:** USA<br>**Ethics:** All subjects gave written consent to a protocol approved by the University of Iowa Human Subjects Institutional Review Board.
**Participants**	**Sample size:** SCZ 18; HC 13<br>**Age (Mean ± SD):** SCZ 32.5 ± 11; HC 26.5 ± 6.4<br>**Gender (F/M):** SCZ 5/13; HC 7/6<br>**Handedness (L/R):** SCZ 0/18; HC 0/13<br>**Ethnicity:** Not reported<br>**Inclusion criteria:**<br>1. A diagnosis of SCZ based on DSM-IV.<br>2. Drug naive or had been medication-free for 3 weeks before the study.<br>3. HCs were only included if they had no history of psychiatric, neurological, or general medical illnesses.<br>**Exclusion criteria:** -
**Assessment**	**Paradigm class:** PET<br>**Equipment properties:** Quantitative PET blood flow data were acquired on a GE 4096-plus whole-body scanner.
**Authors' conclusion**	"Thus, the decreased flow in these regions in our patients may reflect a defect in the activity of mirror neurons in schizophrenia."
**Notes**	**Funding source:** University of Iowa (MH40856, MH60990, MH19113, and MHCRC43271).<br>**Conflicts of interest:** Not reported
**Methodological and reporting quality**	**Overall judgment:** Fair quality<br>**Notes:** The sample size was less than the specified minimum. The definitions, inclusion, and exclusion criteria used to identify or select cases and controls were probably not valid and reliable. Controls were not matched with cases for age or gender.

Andrews 2015 (2)

**Methods**	**Setting:** Outpatients at the Alfred hospital<br>**Country:** Australia<br>**Ethics:** Ethical approval was obtained from the Alfred hospital and Monash University. Informed consent was provided by all participants.
**Participants**	**Sample size:** SCZ 19 (12 SCZ, 7 schizoaffective); HC 19<br>**Age (Mean ± SD):** SCZ 44.16 ± 11.49; HC 37.84 ± 13.07<br>**Gender (F/M):** SCZ 8/11; HC 10/9<br>**Handedness (L/R):** Not reported<br>**Ethnicity:** Not reported<br>**Inclusion criteria:**<br>1. Refrain from substance use, including caffeine or alcohol, for 12 hours before participating.<br>2. A diagnosis of SCZ or schizoaffective disorder, confirmed by the administration of the MINI International Neuropsychiatric Interview.<br>**Exclusion criteria:**<br>1. History of seizures, serious head injury, or neurosurgery.<br>2. Metal implanted in the cranium.<br>3. Pregnant women.<br>4. Current substance dependence.<br>5. History of psychiatric (except SCZ for clinical participants) or neurological disorder.<br>6. Significant extrapyramidal side effects (i.e., an overall score of <10 on the Simpson-Angus Scale and Abnormal Involuntary Movements Scale).
**Assessment**	**Paradigm class:** TMS/EMG/EEG<br>**Equipment properties:** TMS Device: Magstim-200 stimulator; Target: primary motor cortex (M1); MEP: right first dorsal interosseus (FDI); RMT: The minimum stimulation intensity required, to elicit a ≥50 µV MEP in at least 3/5 consecutive trials.<br>EEG Device: Neuroscan Synamps System; SR: 1000 Hz; Channels: -; Bandpass filter: 0.1–100 Hz; Cap: (Neuroscan; System: International 10-20; EOG: above, below, right, and left of the eyes.
**Authors' conclusion**	"Levels of mu suppression and motor resonance were not significantly different between groups. These findings indicate that in stable outpatients with SCZ, mirror system functioning is intact, and therefore their social cognitive difficulties may be caused by alternative pathophysiology."
**Notes**	**Funding source:** P.G.E. was supported by the Clinical Research Fellowship Grant from the National Health and Medical Research Council (NHMRC). K.E.H. was supported by an NHMRC fellowship. P.B.F. was supported by an NHMRC Practitioner Fellowship grant.<br>**Conflicts of interest:** P.B.F. has received equipment for research from Medtronic, MagVenture A/S, and Brainsway Ltd and research funding from Cervel Neurotech. The other authors declare no conflicts of interest.
**Methodological and reporting quality**	**Overall judgment:** Good quality<br>**Notes:** It was not reported if controls were matched with cases for handedness.

Bagewadi 2019 (3)

**Methods**	**Setting:** A tertiary care neuropsychiatric hospital<br>**Country:** India<br>**Ethics:** All participants provided written informed consent that was approved by the Institute's Ethics Committee. Both the MD dissertation protocols were reviewed and approved by this Ethics Committee.
**Participants**	**Sample size:** SCZ 30; HC 28<br>**Age (Mean ± SD):** SCZ 28.6 ± 4.5; HC 26.5 ± 4.3<br>**Gender (F/M):** SCZ 15/24; HC 13/15<br>**Handedness (L/R):** SCZ 0/30; HC 0/28<br>**Ethnicity:** Asian<br>**Inclusion criteria:**<br>1. Diagnosis of SCZ based on DSM-IV as evaluated by a qualified psychiatrist and confirmed using the Mini International Neuropsychiatric Interview.<br>2. Right handedness.<br>3. No contraindications to participating in a TMS study.<br>4. No substance use disorders (except for nicotine) as determined by clinical interview.<br>5. No clinically diagnosable or self-reported visual or auditory impairment.<br>6. No current pregnancy/postpartum state.<br>7. HC subjects were screened to rule out any history of a psychiatric diagnosis using the Mini International Neuropsychiatric Interview – Screening instrument or a family history of psychosis in a first-degree relative.<br>**Exclusion criteria:** -
**Assessment**	**Paradigm class:** TMS/EMG<br>**Equipment properties:** Device: MagPro R30; Target: left motor cortex hand area; MEP: right first dorsal interosseus (FDI); RMT: The minimum stimulation intensity required, to elicit a ≥50 µV MEP in at least 6/10 consecutive trials; SI_{1mV}: The minimum stimulation intensity required, to elicit a ≥1 mV MEP in at least 6/10 consecutive trials.
**Authors' conclusion**	"Providing a context to the action modulates MNS-activity. This modulation is diminished in SCZ patients, suggestive of a diminished sensorimotor associative learning process. This novel, ecologically valid paradigm to tap into the MNS may serve as a neural-marker of social cognition performance in SCZ."
**Notes**	**Funding source:** UMM is supported by the Wellcome Trust/DBT India Alliance Early Career Fellowship, Grant/Award Number: IA/E/12/1/500755.<br>**Conflicts of interest:** UMM is one of the Associate Editors at SCZ Research and receives an honorarium from Elsevier for this service. None of the other authors report any potential conflict of interest.
**Methodological and reporting quality**	**Overall judgment:** Good quality<br>**Notes:** Controls were not matched with cases for gender.

Brown 2016 (4)

**Methods**	**Setting:** LWL University Hospital Bochum<br>**Country:** Germany<br>**Ethics:** The study was approved by the local ethics committee and was performed following the Declaration of Helsinki. All participants gave written consent to participate in the study.
**Participants**	**Sample size:** SCZ 17; HC 17<br>**Age (Mean ± SD):** SCZ 41.71 ± 10.98; HC 38.29 ± 12.10<br>**Gender (F/M):** SCZ 8/9; HC 8/9<br>**Handedness (L/R):** SCZ 0/17; HC 0/17<br>**Ethnicity:** White<br>**Inclusion criteria:**<br>1. A diagnosis of SCZ based on ICD-10, made by a psychiatrist.<br>2. Right-handedness.<br>3. No history of neurological injury.<br>4. No obvious motor deficits.<br>5. No substance dependence/abuse.<br>6. No change in antipsychotic medication within the last 6 months.<br>7. HCs were only included if they had no history of psychiatric illness, no neurological injury, and no deficits in motor functioning.<br>**Exclusion criteria:** -
**Assessment**	**Paradigm class:** EEG<br>**Equipment properties:** Device: Brain Products BrainAmp system; SR: 512 Hz; Channels: 32; Bandpass filter: 1–100 Hz; Cap: EasyCap; System: -; EOG: one below the left eye.
**Authors' conclusion**	"The apparent trend in the reduced mu suppression in SCZ patients is in line with our main hypothesis, and with other work demonstrating reduced activity in the MNS in SCZ. However, the lack of significance found in this study implies that this impairment may not be as substantial as some others have suggested. Our findings suggest that reward may play a substantial role in the degree of MNS activity in SCZ, as patients showed an intact reward-related modulation of the mu suppression during action observation."
**Notes**	**Funding source:** Not reported<br>**Conflicts of interest:** No potential conflict of interest was reported by the authors.
**Methodological and reporting quality**	**Overall judgment:** Good quality<br>**Notes:** The sample size was less than the specified minimum.

Choe 2018 (5)

**Methods**	**Setting:** Seoul Youth Clinic<br>**Country:** Korea<br>**Ethics:** Not reported
**Participants**	**Sample size:** SCZ 26 (all first-episode psychosis); HC 26<br>**Age (Mean ± SD):** SCZ 23.77 ± 5.95; HC 22.62 ± 5.29<br>**Gender (F/M):** SCZ 14/12; HC 15/11<br>**Handedness (L/R):** SCZ 3/23; HC 2/24<br>**Ethnicity:** Asian<br>**Inclusion criteria:** Not reported<br>**Exclusion criteria:** Not reported
**Assessment**	**Paradigm class:** rs-fMRI<br>**Equipment properties:** SMF = 3 T; Scanner = Siemens Trio; Sequence = GE EPI, TE = -; TR = -; Slice thickness = -; Matrix size = -; FA = -; Volumes = -.
**Authors' conclusion**	"Group comparisons using independent t-tests indicated that several connections within the MNS network showed altered connectivity in FEP patients compared to controls."
**Notes**	**Funding source:** This research was supported by the Brain Research Program through the National Research Foundation of Korea (NRF) funded by the Ministry of Science, ICT & Future Planning (Grant no. 2017M3C7A1029610).<br>**Conflicts of interest:** No potential conflict of interest was reported by the authors.
**Methodological and reporting quality**	**Overall judgment:** Poor quality<br>**Notes:** It was not reported if controls were screened for other psychiatric or neurological disorders. The definitions, inclusion, and exclusion criteria used to identify or select cases and controls were not reported. Cases and/or controls were not randomly selected from those eligible. We cannot determine if the measures of the assessment paradigm are valid and reliable for this review's subject. It was not reported if ethical issues were considered in the study design.

Das 2011 (6)

**Methods**	**Setting:** Royal North Shore Hospital<br>**Country:** Australia<br>**Ethics:** A joint hospital and university ethics committee approved the study, and after a complete description of the study to the subjects, all participants provided written informed consent.
**Participants**	**Sample size:** SCZ 20; HC 19<br>**Age (Mean ± SD):** SCZ 34.5 ± 8.4; HC 33.5 ± 8.4<br>**Gender (F/M):** SCZ 0/20; HC 0/19<br>**Handedness (L/R):** SCZ 0/20; HC 0/19<br>**Ethnicity:** White<br>**Inclusion criteria:**<br>1. Diagnosis of SCZ based on the Structured Clinical Interview for DSM-IV.<br>**Exclusion criteria:**<br>1. History of neurological disease, closed head injury, or a medical disorder necessitating treatment<br>2. A twelve-month history of substance misuse or dependence.<br>3. Patients had no additional Axis-I or Axis-II psychiatric diagnoses.
**Assessment**	**Paradigm class:** T-fMRI<br>**Equipment properties:** SMF = 3 T; Scanner = Siemens Trio; Sequence = GE EPI, TE = 35 ms; TR = 3 s; Slice thickness = 5 mm; Matrix size = 64 x 64; FA = 90°; Volumes = 224.
**Authors' conclusion**	"Patients with schizophrenia had significantly diminished activity in the right superior temporal gyrus (STG) at the temporoparietal junction (TPJ) and bilaterally within the inferior frontal gyri (IFG)."
**Notes**	**Funding source:** Funding for this study was provided by NHMRC Program Grant 510135. NSW Institute of Psychiatry Fellowship supported Dr. Henderson.<br>**Conflicts of interest:** Dr. Pritha Das, Dr. Carissa Coulston, and Associate Professor Lagopoulos have no interest to declare. Dr. Henderson has been supported by a Pfizer Neuroscience fellowship in 2009. Professor Gin Malhi has served on many international and national pharmaceutical advisory boards, received funding for research, and has been in receipt of honoraria for talks at sponsored meetings worldwide involving the following companies: AstraZeneca, Eli Lilly, Janssen-Cilag, Organon, Pfizer, and Wyeth.
**Methodological and reporting quality**	**Overall judgment:** Good quality<br>**Notes:** We cannot determine if the research question or objective is appropriate for this review's subject.

ElShahawi 2020 (7)

**Methods**	**Setting:** Inpatient department and outpatient clinics of the institute of psychiatry<br>**Country:** Egypt<br>**Ethics:** The nature of the study was discussed with each patient and written informed consent was obtained from all patients before participation in the study. This study was approved by the Ain Shams University Hospital Ethics Committee.
**Participants**	**Sample size:** SCZ 15; HC 15<br>**Age (Mean ± SD):** SCZ 29 ± 8; HC 30 ± 8<br>**Gender (F/M):** SCZ 3/12; HC 3/12<br>**Handedness (L/R):** Not reported<br>**Ethnicity:** White<br>**Inclusion criteria:**<br>1. A diagnosis of SCZ based on DSM-IV.<br>2. Age of 18-40 years.<br>3. IQ>70.<br>4. HCs were only included if they didn't have any Axis I psychiatric or chronic medical illnesses, DSM-IV-TR substance abuse in the past month or substance dependence in the past 3 months (except for nicotine addiction), any first-degree relatives with psychosis, any metal implants, or any other contraindications for MRI.<br>**Exclusion criteria:**<br>1. Having a concurrent Axis I psychiatric disorder or a chronic medical illness.<br>2. Having DSM-IV-TR substance abuse in the past month or substance dependence in the past 3 months (except for nicotine addiction).<br>3. History of any metal implants or any other contraindications for MRI.<br>4. Received ECT sessions within 3 months before the study.
**Assessment**	**Paradigm class:** DWI/DTI<br>**Equipment properties:** SMF = 1.5 T; Scanner = Achieva Philips; Sequence = SE EPI, TE = 68.7 ms; TR = 8 s; Slice thickness = 2 mm; Matrix size = 80 x 78; Volumes = 224.
**Authors' conclusion**	"Concerning DTI findings, we investigated three regions of interest (ROIs); anterior cingulate gyrus (ACC), inferior parietal lobe (IPL), and premotor area (PMA)- which are believed to be parts of the MNS. Our primary findings observed overall higher diffusivity in apparent diffusion coefficient (ADC) "trace" in all three areas on both sides in schizophrenia patients compared to healthy controls."
**Notes**	**Funding source:** No sources of support were provided for this study.<br>**Conflicts of interest:** No potential conflict of interest was reported by the authors.
**Methodological and reporting quality**	**Overall judgment:** Fair quality<br>**Notes:** The sample size was less than the specified minimum. It was not reported if controls were matched with cases for handedness.

Enticott 2008 (8)

**Methods**	**Setting:** Not reported<br>**Country:** Australia<br>**Ethics:** This project was approved by the ethics committees of Alfred and Monash University. All participants provided informed consent.
**Participants**	**Sample size:** SCZ 15 (4 Schizoaffective); HC 15<br>**Age (Mean ± SD):** SCZ 41.80 ± 8.26; HC 35.20 ± 10.13<br>**Gender (F/M):** SCZ 3/12; HC 6/9<br>**Handedness (L/R):** Not reported<br>**Ethnicity:** White<br>**Inclusion criteria:**<br>1. A diagnosis of either SCZ or schizoaffective disorder according to DSM-IV.<br>2. Free of benzodiazepines and anticholinergics for a minimum of four weeks.<br>3. 3. No significant extrapyramidal side-effects (i.e., the overall score of < 10 on the Simpson–Angus Scale and Abnormal Involuntary Movements Scale).<br>**Exclusion criteria:** -
**Assessment**	**Paradigm class:** TMS/EMG<br>**Equipment properties:** Device: Magstim-200 stimulator; Target: left primary motor cortex (M1); MEP: right abductor pollicis brevis; RMT: The minimum stimulation intensity required, to elicit a ≥50 µV MEP in at least 3/5 consecutive trials.
**Authors' conclusion**	"While patients demonstrated no abnormalities in cortical excitability, motor facilitation during action observation, putatively reflecting mirror neuron activity, was reduced in SCZ."
**Notes**	**Funding source:** Funding for this study was provided by Monash University's Faculty of Medicine, Nursing, and Health Sciences. PF was supported by an NHMRC Practitioner Fellowship. PJ was supported by an NHMRC Clinical Training Fellowship.<br>**Conflicts of interest:** No potential conflict of interest was reported by the authors.
**Methodological and reporting quality**	**Overall judgment:** Poor quality<br>**Notes:** The sample size was less than the specified minimum. It was not reported if controls were screened for other psychiatric or neurological disorders. The definitions, inclusion, and exclusion criteria used to identify or select cases and controls were probably not valid and reliable. Controls were not matched with cases for gender. It was not reported if controls were matched with cases for handedness.

Ferri 2014 (9)

**Methods**	**Setting:** Outpatient services at Chieti Mental Health Department<br>**Country:** Italy<br>**Ethics:** The Ethics Committee of the University of Chieti approved the study. Written informed consent was obtained from all participants after a full explanation of the procedure of the study, in line with the Declaration of Helsinki.
**Participants**	**Sample size:** SCZ 22; HC 22<br>**Age (Mean ± SD):** SCZ 27.45 ± 5.07; HC 28 ± 3.77<br>**Gender (F/M):** SCZ 8/14; HC 10/12<br>**Handedness (L/R):** Not reported<br>**Ethnicity:** White<br>**Inclusion criteria:**<br>1. Diagnosis of SCZ based on DSM-IV.<br>2. HCs were only included if they didn't have a personal history of axis I/II disorders or a history of psychosis in first-degree relatives.<br>**Exclusion criteria:**<br>1. Significant medical or neurological illness.<br>2. Substance abuse or dependence in the previous 6 months.<br>3. IQ <85.
**Assessment**	**Paradigm class:** T- fMRI<br>**Equipment properties:** SMF = 1.5 T; Scanner = Philips Achieva scanner; Sequence = GE EPI, TE = 50 ms; TR = 2.4 s; Slice thickness = 4 mm; Matrix size = 64 x 64; FA = -; Volumes = 216.
**Authors' conclusion**	"In conclusion, our study supports previous evidence of poorer mirror neuron-driven embodied simulation (mnES) during action observation in SCZ. Moreover, it goes further by showing that emotional cues might allow patients to recover mnES, at least in part. However, their understanding of the emotional components of others' actions will likely remain deficient."
**Notes**	**Funding source:** This work was supported by the EU grant TESIS to Vittorio Gallese.<br>**Conflicts of interest:** Not reported
**Methodological and reporting quality**	**Overall judgment:** Fair quality<br>**Notes:** Controls were not matched with cases for gender. It was not reported if controls were matched with cases for handedness.

Guo 2014 (10)

**Methods**	**Setting:** National Taiwan University Hospital<br>**Country:** Taiwan<br>**Ethics:** Written informed consent was obtained from all individual participants, and all of the research procedures and ethical guidelines were followed under the Institutional Review Board (IRB) of the National Taiwan University Hospital.
**Participants**	**Sample size:** SCZ 69; HC 62<br>**Age (Mean ± SD):** SCZ 31.95 ± 9.60; HC 29.87 ± 8.62<br>**Gender (F/M):** SCZ 34/35; HC 37/25<br>**Handedness (L/R):** SCZ 2/67; HC 1/61<br>**Ethnicity:** Asian<br>**Inclusion criteria:**<br>1. Diagnosis of SCZ based on DSM-IV.<br>2. HCs were only included if they didn't have a personal history of SCZ and other Axis I disorders based on DSM-IV.<br>**Exclusion criteria:**<br>1. Presence of DSM-IV Axis I diagnoses of other disorders such as bipolar disorder.<br>2. History of any substance dependence.<br>3. History of any neurological disorders.<br>4. History of clinically significant head trauma.
**Assessment**	**Paradigm class:** rs-fMRI<br>**Equipment properties:** SMF = 3 T; Scanner = TIM Trio, Siemens; Sequence = GE EPI, TE = 24 ms; TR = 2.0 s; Slice thickness = 3 mm; Matrix size = 64 x 64; FA = 90°; Volumes = -.
**Authors' conclusion**	"In summary, our results using resting-state fMRI to analyze functional connectivity changes in medicated SCZ patients have shown that by far the most affected pathway primarily includes parietal components of the medial cortical and mirror neuron systems involved in many self-processing and other sensory, cognitive, and executive functions known to be impaired in this disorder."
**Notes**	**Funding source:** National Nature Science Foundation of China, Grant numbers: 10901049, 11271121. Key Laboratory of Computational and Stochastic Mathematics and Its Application of Hunan province, Grant number: 11K038. Hunan Normal University, Grant number: ET11001.<br>**Conflicts of interest:** JF is a Royal Society Wolfson Research Merit award holder.
**Methodological and reporting quality**	**Overall judgment:** Good quality<br>**Notes:** Controls were not matched with cases for gender.

He 2021 (11)

**Methods**	**Setting:** Department of Psychiatry and Psychotherapy at the Philipps University of Marburg<br>**Country:** Germany<br>**Ethics:** All participants gave written informed consent prior to participation in the experiment and were compensated monetarily. The study was approved by the ethics committee of the School of Medicine, Philipps University Marburg.
**Participants**	**Sample size:** SCZ 17; HC 18<br>**Age (Mean ± SD):** SCZ 33.12 ± 12.35; HC 31.94 ± 10.21<br>**Gender (F/M):** SCZ 4/13; HC 5/13<br>**Handedness (L/R):** SCZ 1/16; HC 1/17<br>**Ethnicity:** White<br>**Inclusion criteria:**<br>1. Diagnosis of SCZ or schizoaffective disorder based on ICD-10.<br>2. Normal or corrected-to-normal vision and hearing.<br>**Exclusion criteria:**<br>1. Brain injury and neurological or other medical diseases.
**Assessment**	**Paradigm class:** T-fMRI<br>**Equipment properties:** SMF = 3 T; Scanner = Siemens MRT Trio; Sequence = GE EPI, TE = 30 ms; TR = 2 s; Slice thickness = 4 mm; Matrix size = 64 x 64; FA = 90°; Volumes = 425.
**Authors' conclusion**	"With regard to the patients, we observed normal neural processing of non-social content in the gesture modality, supporting a previous study which reported intact mirror neuron activity in schizophrenia."
**Notes**	**Funding source:** This research project is supported by a grant from the 'Von-Behring- Ro ň tgen-Stiftung' (project no. 59-0002 and 64-0001) and by the 'Deutsche Forschungsgemeinschaft' (project no. DFG: STR1146/11-2 & KI588/6-2, HE8029/2-1, and CRC/TRR 135/2 project A3, number: 222641018). The study was also supported by the Core Facility Brain Imaging, Faculty of Medicine, University of Marburg, Rudolf-Bultmann- Str. 9, 35039, Marburg, Germany.<br>**Conflicts of interest:** No potential conflict of interest was reported by the authors.
**Methodological and reporting quality**	**Overall judgment:** Good quality<br>**Notes:** The definitions, inclusion, and exclusion criteria used to identify or select cases and controls were probably not valid and reliable (did not evaluate substance abuse/dependence).

Horan 2014-1 (12)

**Methods**	**Setting:** Outpatient clinics at the VA Greater Los Angeles Healthcare System<br>**Country:** USA<br>**Ethics:** All participants were evaluated for their capacity to give informed consent and provided written informed consent after all procedures were fully explained, according to procedures approved by the institutional review boards at the University of California, Los Angeles (UCLA), and the Greater Los Angeles VA Health Care System.
**Participants**	**Sample size:** SCZ 23; HC 23<br>**Age (Mean ± SD):** SCZ 46.5 ± 11.1; HC 46.7 ± 6.9<br>**Gender (F/M):** SCZ 6/17; HC 7/16<br>**Handedness (L/R):** Not reported<br>**Ethnicity:** White 67.4%, African American 30.4%, Hispanic 2.2%.<br>**Inclusion criteria:**<br>1. Diagnosis of SCZ based on DSM-IV.<br>**Exclusion criteria:**<br>1. The exclusion criteria for patients included:<br>o Substance abuse or dependence in the last 6 months.<br>o IQ<70.<br>o History of loss of consciousness for >1 h or neurological disorder.<br>2. Exclusion criteria for the control participants included:<br>o History of SCZ or other psychotic disorders, substance abuse in the last 6 months.<br>o Avoidant, paranoid, schizoid, and schizotypal disorders.<br>o History of loss of consciousness for >1 h or significant neurological disorder.<br>o SCZ or other psychotic disorder in a first-degree relative.
**Assessment**	**Paradigm class:** T-fMRI<br>**Equipment properties:** SMF = 3 T; Scanner = Siemens Trio; Sequence = GE EPI, TE = 30 ms; TR = 2 s; Slice thickness = 4 mm; Matrix size = 64 x 64; FA = 75°; Volumes = 116.
**Authors' conclusion**	"Although it has been hypothesized that impaired neural mirroring is associated with schizophrenia, our findings are not consistent with this notion."
**Notes**	**Funding source:** Support for this study came from a VA Career Development Award (William P. Horan, Ph.D.), NIMH Grants MH065707, and MH43292 (Michael F. Green, Ph.D.), and the Attias Family Foundation (Marco Iacoboni, MD Ph.D.).<br>**Conflicts of interest:** Dr. Green reports having received consulting fees from Abbott Laboratories, Amgen, Cypress, Lundbeck, and Teva. He has received speaking fees from Otsuka and Sunovion. The rest of the authors report no biomedical financial interests or potential conflicts of interest.
**Methodological and reporting quality**	**Overall judgment:** Good quality<br>**Notes:** We cannot determine if controls were matched with cases for handedness.

Horan 2014-2 (13)

**Methods**	**Setting:** Outpatient clinics at the VA Greater Los Angeles Healthcare System<br>**Country:** USA<br>**Ethics:** Participants were provided written informed consent after all procedures had been fully explained, according to procedures approved by the institutional review boards at the VA Greater Los Angeles Healthcare System and the University of California, Los Angeles (UCLA).
**Participants**	**Sample size:** SCZ 32; HC 26<br>**Age (Mean ± SD):** SCZ 47.9 ± 9.6; HC 44.4 ± 7.9<br>**Gender (F/M):** SCZ 6/26; HC 7/19<br>**Handedness (L/R):** SCZ 6/26; HC 5/21<br>**Ethnicity:** White 56.3%, African American 31.3%, Hispanic 6.3%, Asian 3.1%.<br>**Inclusion criteria:**<br>1. Diagnosis of SCZ based on DSM-IV.<br>2. No hospitalizations in the past 3 months.<br>3. No changes in the living situation in the past 2 months.<br>4. No medication changes in the past 6 weeks.<br>**Exclusion criteria:**<br>1. The exclusion criteria for patients included:<br>o Substance abuse or dependence in the last 6 months.<br>o IQ<70.<br>o History of loss of consciousness for >1 h or neurological disorder.<br>2. Exclusion criteria for the control participants included:<br>o History of SCZ or other psychotic disorders, substance abuse in the last 6 months.<br>o Avoidant, paranoid, schizoid, and schizotypal disorders.<br>o History of loss of consciousness for >1 h or significant neurological disorder.<br>o SCZ or other psychotic disorder in a first-degree relative.
**Assessment**	**Paradigm class:** EEG<br>**Equipment properties:** Device: ActiveTwo BioSemi system; SR: 1024 Hz; Channels: 64; Bandpass filter: 1–100 Hz; Cap: Cortech Solutions; System: International 10–20; EOG: two 1 cm above and below the left eye, one 1 cm to the left of the left eye, and one 1 cm to the right of the right eye.
**Authors' conclusion**	"The SCZ group showed generally intact modulation of MNS functioning at the electrophysiological level, despite self-reporting empathic disturbances. The disturbances commonly seen on self-report, performance may largely reflect difficulties with higher-level inferential processes about others' emotions, rather than a basic incapacity to share in these experiences."
**Notes**	**Funding source:** Support for this study came from a VA Career Development Award (to W.P.H.) and NIMH Grant Nos. MH065707 and MH43292 (M.F.G.).<br>**Conflicts of interest:** M.F.G. reports having received consulting fees from Abbott Laboratories, Amgen, Cypress, Lundbeck, and Teva. He has received speaking fees from Otsuka and Sunovion. The rest of the authors report no biomedical financial interests or potential conflicts of interest.
**Methodological and reporting quality**	**Overall judgment:** Good quality<br>**Notes:** Controls were not matched with cases for gender.

Horan 2016 (14)

**Methods**	**Setting:** Outpatient clinics at the VA Greater Los Angeles Healthcare System<br>**Country:** USA<br>**Ethics:** All participants provided written informed consent to participate.
**Participants**	**Sample size:** SCZ 21; HC 21<br>**Age (Mean ± SD):** SCZ 48.2 ± 10.4; HC 46.5 ± 7.1<br>**Gender (F/M):** SCZ 6/15; HC 7/14<br>**Handedness (L/R):** SCZ 2/19; HC 3/18<br>**Ethnicity:** White 44%, African American 38%, Hispanic 16.5%, Asian 1.5%.<br>**Inclusion criteria:**<br>1. Diagnosis of SCZ based on DSM-IV.<br>2. Age of 18-60.<br>3. No current substance use disorder.<br>4. No identifiable neurological disorder.<br>5. Sufficient English fluency.<br>6. HCs were only included if they didn't have a history of psychosis in first-degree relatives.<br>**Exclusion criteria:**<br>1. History of psychotic disorder, bipolar disorder, recurrent depression, dysthymia, or substance dependence disorder.<br>2. Avoidant, paranoid, schizoid, or schizotypal personality disorders.<br>3. History of loss of consciousness >1 h.
**Assessment**	**Paradigm class:** T-fMRI<br>**Equipment properties:** SMF = 3 T; Scanner = TIM Trio, Siemens; Sequence = -, TE = -; TR = -; Slice thickness = -; Matrix size = -; FA = -; Volumes = -.
**Authors' conclusion**	"Although individuals with schizophrenia showed relatively intact sensitivity to the pain of others, their ability to process this information in a manner that promotes adaptive responding appears to be impaired."
**Notes**	**Funding source:** Support for this study came from a VA Career Development Award (William P. Horan, PhD.) and NIMH Grants MH065707 and MH43292 (Michael F. Green, Ph.D.).<br>**Conflicts of interest:** Dr. Green reports having received consulting fees from AbbVie, DSP, Forum, Mnemosyne (scientific board), Takeda, Roche. He has received grant funding from Amgen and Forum. The rest of the authors report no biomedical financial interests or potential conflicts of interest.
**Methodological and reporting quality**	**Overall judgment:** Fair quality<br>**Notes:** We cannot determine if the research question or objective is appropriate for this review's subject.

Kato 2011 (15)

**Methods**	**Setting:** Not reported<br>**Country:** Japan<br>**Ethics:** All subjects gave written informed consent. This study was approved by the ethics committee of Tokyo Dental College following the Declaration of Helsinki protocols.
**Participants**	**Sample size:** SCZ 15; HC 15<br>**Age (Mean ± SD):** SCZ 33.4 ± 6.6; HC 32.7 ± 5.6<br>**Gender (F/M):** SCZ 8/7; HC 7/8<br>**Handedness (L/R):** SCZ 0/15; HC 0/15<br>**Ethnicity:** Asian<br>**Inclusion criteria:**<br>1. Antipsychotic-free for at least six months.<br>2. SCZ diagnosis based on DSM-IV-TR confirmed by the SCID-I interview (Structured Interview for DSM-IV).<br>3. Having normal or normal-corrected visual acuity.<br>4. Right-handedness according to the Edinburgh Scale.<br>**Exclusion criteria:** -
**Assessment**	**Paradigm class:** MEG<br>**Equipment properties:** Brain magnetic signals were measured with a whole-scalp 306-channel neuromagnetometer equipped with 204 planar gradiometers and 102 magnetometers (Vectorview).
**Authors' conclusion**	"Our findings demonstrate that untreated patients with SCZ exhibit aberrant mirror neuron system function based on the right inferior parietal cortex, which is characterized by dysfunction of gamma-synchronization in the right parietal lobe during observation of biological motion."
**Notes**	**Funding source:** This work was supported by a grant (HRC6A02) from the Ministry of Education, Culture, Sports, Science and Technology (MEXT) of Japan to M.K. and by a Grant-in-Aid for Scientific Research on Innovative Areas: Prediction and Decision Making (23120009). The funders had no role in study design, data collection, and analysis, decision to publish, or preparation of the manuscript.<br>**Conflicts of interest:** No potential conflict of interest was reported by the authors.
**Methodological and reporting quality**	**Overall judgment:** Poor quality<br>**Notes:** The sample size was less than the specified minimum. It was not reported if controls were screened for other psychiatric or neurological disorders. The definitions, inclusion, and exclusion criteria used to identify or select cases and controls were probably not valid and reliable.

Lee 2014 (16)

**Methods**	**Setting:** Psychiatric outpatient clinic<br>**Country:** Korea<br>**Ethics:** The study was approved by the institutional review board, and written informed consent was obtained from all participants.
**Participants**	**Sample size:** SCZ 15; HC 16<br>**Age (Mean ± SD):** SCZ 36.7 ± 8.1; HC 36.8 ± 6.3<br>**Gender (F/M):** SCZ 6/9; HC 6/10<br>**Handedness (L/R):** SCZ 0/15; HC 0/16<br>**Ethnicity:** Asian<br>**Inclusion criteria:**<br>1. Diagnosis of SCZ based on Structural Clinical Interview for DSM-IV.<br>2. HCs were only included if they didn't have a personal history of psychiatric disorders.<br>3. Right-handedness.<br>**Exclusion criteria:**<br>1. Presence of a neurological or significant medical illness.<br>2. Current or past substance abuse or dependence.
**Assessment**	**Paradigm class:** T-fMRI<br>**Equipment properties:** SMF = 3 T; Scanner = Intra Achieva; Philips; Sequence = GE EPI, TE = 30 ms; TR = 2.5 s; Slice thickness = 3.5 mm; Matrix size = 128 x 128; FA = 90°; Volumes = -.
**Authors' conclusion**	"Our findings of widespread cortical changes during facial expression in patients included most regions of the MNS. Our study, directly addressing blunted affect using facial expression, suggests a new perspective that disturbance of the MNS may play an important role in the manifestation of blunted affect in SCZ."
**Notes**	**Funding source:** This work was supported by a National Research Foundation of Korea (NRF) grant funded by the Korean government (MEST; No. 2011-0015859).<br>**Conflicts of interest:** No potential conflict of interest was reported by the authors.
**Methodological and reporting quality**	**Overall judgment:** Poor quality<br>**Notes:** We cannot determine if the research question or objective is appropriate for this review's subject. The sample size was less than the specified minimum. We cannot determine if the measures of the assessment paradigm are valid and reliable for this review's subject.

McCormick 2012 (17)

**Methods**	**Setting:** Inpatient unit at the University of Iowa Hospitals and Clinics<br>**Country:** USA<br>**Ethics:** This study was approved by the Institutional Review Board at the University of Iowa, and written informed consent was obtained from all subjects after the procedures had been fully explained.
**Participants**	**Sample size:** SCZ 16 (14 SCZ, 1 schizoaffective, 1 delusional disorder); HC 16<br>**Age (Mean ± SD):** SCZ 37.0 ± 9.8; HC 36.6 ± 9.7<br>**Gender (F/M):** SCZ 2/14; HC 2/14<br>**Handedness (L/R):** SCZ 1/13/2 other; HC 0/16<br>**Ethnicity:** Not reported<br>**Inclusion criteria:**<br>1. Diagnosis of SCZ spectrum disorders (i.e., SCZ, schizoaffective disorder, delusional disorder) by board-certified psychiatrists using DSM-IV-TR.<br>2. HCs were only included if they didn't have a family history of SSD, other psychotic disorder, or autism, history of treatment with psychotropic medications including benzodiazepines for a psychiatric disorder, substance use within the past three months, and history of seizure, head injury with loss of consciousness greater than five minutes, or other neurological disorder.<br>**Exclusion criteria:**<br>1. Recent use of a long-acting benzodiazepine and self-reported drug.<br>2. Alcohol abuse or dependence within the past three months.
**Assessment**	**Paradigm class:** EEG<br>**Equipment properties:** Device: Cadwell Easy II; SR: 200 Hz; Channels: 19; Bandpass filter: 1–70 Hz; Cap: -; System: International 10-20; EOG: -.
**Authors' conclusion**	"EEG was used to assess mu suppression as a non-invasive measure of mirror neuron activity. SCZ subjects with active psychosis were found to have greater left-sided mu suppression or greater mirror neuron activity, which correlated to greater psychotic symptoms. The present research showed that mirror neuron activity is elevated among patients with SCZ and active psychosis."
**Notes**	**Funding source:** Not reported<br>**Conflicts of interest:** Not reported
**Methodological and reporting quality**	**Overall judgment:** Fair quality<br>**Notes:** The sample size was less than the specified minimum. Cases and/or controls were not randomly selected from those eligible.

Mehta 2014 (18)

**Methods**	**Setting:** Inpatient and outpatient services of the National Institute of Mental Health & Neurosciences, Bangalore<br>**Country:** India<br>**Ethics:** The study was conducted at the National Institute of Mental Health & Neurosciences (NIMHANS), Bangalore, and the institute's ethics committee approved the study. Also written informed consent was obtained.
**Participants**	**Sample size:** SCZ 54 (33 antipsychotics naive, 21 medicated); HC 45<br>**Age (Mean ± SD):** SCZ Antipsychotic naive: 33.60 ± 9.74 Medicated: 29.19 ± 6.60; HC 30.68 ± 30.68 ± 9.57<br>**Gender (F/M):** SCZ Antipsychotic naive: 15/17 Medicated: 12/9; HC 17/19<br>**Handedness (L/R):** SCZ 0/54; HC 0/45<br>**Ethnicity:** Asian<br>**Inclusion criteria:**<br>1. Right-handedness.<br>2. A diagnosis of SCZ based on DSM-IV and confirmed using the Mini-International Neuropsychiatric Interview (MINI).<br>3. HCs were screened to rule out Axis-1 psychiatric disorders using MINI-Screening.<br>4. HCs were only included if they didn't have a family history of psychotic disorder in first- and second-degree relatives.<br>**Exclusion criteria:**<br>1. Patients with substance dependence in the previous 6 months (except nicotine).<br>2. Presence of comorbid neurological or medical disorder.<br>3. Clinically diagnosable or self-reported visual or auditory impairment.<br>4. Current pregnancy or postpartum state.<br>5. A score of ≤19 on the Hindi Mental Status Examination.
**Assessment**	**Paradigm class:** TMS/EMG<br>**Equipment properties:** Device: MagPro R30; Target: hand area of the left motor cortex; MEP: right first dorsal interosseous (FDI); RMT: The minimum stimulation intensity required, to elicit a ≥50 µV MEP in at least 3/5 consecutive trials; SI_{1mV}: The minimum stimulation intensity required, to elicit a ≥1 mV MEP in at least 6/10 consecutive trials.
**Authors' conclusion**	"Antipsychotic-naive SCZ patients have poorer mirror neuron activity than medicated patients and HC. These findings suggest a possibility of deficient mirror neuron systems underlying social cognition deficits in SCZ."
**Notes**	**Funding source:** Department of Biotechnology, Ministry of Science & Technology, Government of India (BT/PR14311/ Med/30/470/2010 to U.M.M.); National Institutes of Health—Harvard Clinical and Translational Science Center/Harvard Catalyst (UL1 RR025758), Sidney-Baer Foundation to P.-L.<br>**Conflicts of interest:** Dr. Pascual-Leone serves on the scientific advisory boards for Nexstim, Neuronix, Starlab Neuroscience, Neuroelectrics, and Neosync; and is listed as an inventor on several issued and pending patents on the real-time integration of EEG, and MRI.
**Methodological and reporting quality**	**Overall judgment:** Good quality<br>**Notes:** -

Mitra 2014 (19)

**Methods**	**Setting:** A premier tertiary care psychiatric institute<br>**Country:** India<br>**Ethics:** It was approved by the institute's ethics committee and all the participants had given informed consent for the same.
**Participants**	**Sample size:** SCZ 15; HC 15<br>**Age (Mean ± SD):** SCZ 28.87 ± 6.8; HC 28.01 ± 6.3<br>**Gender (F/M):** SCZ 3/12; HC 3/12<br>**Handedness (L/R):** SCZ 0/15; HC 0/15<br>**Ethnicity:** Asian<br>**Inclusion criteria:**<br>1. Drug naïve or drug-free for at least 4 weeks for oral and 12 weeks for depot medications.<br>2. Right-handedness.<br>3. HCs were only included if they had a score of < 3 on the General Health Questionnaire.<br>**Exclusion criteria:**<br>1. History of any other comorbid psychiatric disorder.<br>2. History of major neurological or medical illness.<br>3. History of drug or alcohol dependence, except nicotine or caffeine use.<br>4. Electroconvulsive therapy in the past 6 months.
**Assessment**	**Paradigm class:** EEG<br>**Equipment properties:** Device: Neurofax EEG-1100K; SR: 512 Hz; Channels: 192; Bandpass filter: 1–30 Hz; Cap: custom-made; System: International 10-5; EOG: right and left eye.
**Authors' conclusion**	"This article reports a replication of the finding of a dysfunctional MNS system in schizophrenic patients compared to normal controls, in an Indian study. We matched the control subjects for education status as well as the mother tongue, which are improvements over the previous studies, thus giving a better picture of the actual differences without being confounded by the cognitive abilities."
**Notes**	**Funding source:** No sources of support were provided for this study.<br>**Conflicts of interest:** No potential conflict of interest was reported by the authors.
**Methodological and reporting quality**	**Overall judgment:** Fair quality<br>**Notes:** The sample size was less than the specified minimum. The definitions, inclusion, and exclusion criteria used to identify or select cases and controls were probably not valid and reliable.

Moehring 2015 (20)

**Methods**	**Setting:** Outpatient clinic of the Department of Psychiatry, Charité University Medicine Berlin, Campus Benjamin Franklin<br>**Country:** Germany<br>**Ethics:** The study protocol was approved by the ethics committee of the Charité University Medicine Berlin, and the study was conducted following the Declaration of Helsinki and its amendments. All subjects gave written informed consent before participating and received monetary reimbursement for their efforts.
**Participants**	**Sample size:** SCZ 15; HC 15<br>**Age (Mean ± SD):** SCZ 35.60 ± 7.7; HC 35.40 ± 7.9<br>**Gender (F/M):** SCZ 4/11; HC 4/11<br>**Handedness (L/R):** SCZ 0/15; 0/15<br>**Ethnicity:** White<br>**Inclusion criteria:**<br>1. Diagnosis of SCZ based on DSM-IV.<br>2. No psychiatric disorder, other than SCZ.<br>3. No drug abuse other than nicotine abuse/dependence.<br>4. Normal or corrected-to-normal vision.<br>5. Right-handedness according to the Edinburgh Handedness Inventory.<br>6. HCs were only included if they didn't meet the criteria of psychiatric disorders according to DSM-IV as determined by semi-structured clinical interviews and didn't have a family history of psychiatric illness, medical or neurological disorders, and current intake of psychotropic drugs.<br>**Exclusion criteria:**<br>1. Current drug abuse.<br>2. History of severe medical, neurological disorder, or electroconvulsive therapy.<br>3. Extrapyramidal motor side effects due to antipsychotic medication within the last 6 months.
**Assessment**	**Paradigm class:** EEG<br>**Equipment properties:** Device: Advanced Neuro Technology; SR: 512 Hz; Channels: 64; Bandpass filter: 0.1-50 Hz; Cap: custom-made; System: International 10-10; EOG: outer canthus of the left eye.
**Authors' conclusion**	"Our data provide robust evidence that pure perception and execution of hand gestures are relatively intact in SCZ. In contrast, visuomotor transformation processes mediated by the MNS seem to be specifically disturbed in SCZ. These results unambiguously demonstrate MNS deficits in SCZ and extend our understanding of the neuronal bases of social dysfunction in this disorder."
**Notes**	**Funding source:** No sources of support were provided for this study.<br>**Conflicts of interest:** No potential conflict of interest was reported by the authors.
**Methodological and reporting quality**	**Overall judgment:** Good quality<br>**Notes:** The sample size was less than the specified minimum.

Okruszek 2018 (21)

**Methods**	**Setting:** Outpatient clinics in the Warsaw area<br>**Country:** Poland<br>**Ethics:** All participants gave written consent before participating in the study. The procedure of the study was approved by the University of Warsaw Ethics Committee.
**Participants**	**Sample size:** SCZ 25; HC 26<br>**Age (Mean ± SD):** SCZ 35.7 ± 6.9; HC 35.3±7.1<br>**Gender (F/M):** SCZ 12/13; HC 14/12<br>**Handedness (L/R):** SCZ 0/25; HC 0/26<br>**Ethnicity:** White<br>**Inclusion criteria:**<br>1. Diagnosis of SCZ based on ICD-10 confirmed by the clinical interview done by a qualified psychiatrist.<br>2. HCs were only included if they didn't have a personal history of psychiatric or neurological treatment or relatives diagnosed with SCZ.<br>3. Normal or corrected-to-normal vision.<br>**Exclusion criteria:**<br>1. Any change of pharmacotherapy during the 2 weeks before the study.<br>2. History of comorbid head trauma.<br>3. History of drug abuse.<br>4. Intellectual disability.
**Assessment**	**Paradigm class:** T-fMRI<br>**Equipment properties:** SMF = 3 T; Scanner = Siemens Magnetom Trio; Sequence = GE EPI, TE = 28 ms; TR = 2.5 s; Slice thickness = 3 mm; Matrix size = 64 x 64; FA = 90°; Volumes = 468.
**Authors' conclusion**	"HCs were significantly more efficient in recognizing each type of action than SCZ patients. At the neural level, the activity of the right posterior superior temporal sulcus (pSTS) was observed to be higher in HCs compared with SCZ patients for communicative vs. individual action processing. Importantly, the increased connectivity of the right pSTS with structures associated with mentalizing (left pSTS) and mirroring networks (left frontal areas) was observed in HCs, but not in SCZ patients, during the presentation of social interactions."
**Notes**	**Funding source:** This work was funded by the University of Warsaw DSM 2014 and 2015 funds. ŁO was supported by the National Science Centre, Poland (UMO-2015/16/T/HS6/00336 and UMO-2016/23/D/HS6/02947) and Foundation for Polish Science Start program. The project was realized with CePT research infrastructure purchased with funds from the European Regional Development Fund as part of the Innovative Economic Operational Programme, 2007–2013.<br>**Conflicts of interest:** Not reported
**Methodological and reporting quality**	**Overall judgment:** Good quality<br>**Notes:** -

Park 2009 (22)

**Methods**	**Setting:** Outpatients recruited from Severance Mental Health Hospital, Yonsei University College of Medicine.<br>**Country:** Korea<br>**Ethics:** Written informed consent was obtained.
**Participants**	**Sample size:** SCZ 15; HC 16<br>**Age (Mean ± SD):** Not reported<br>**Gender (F/M):** Not reported<br>**Handedness (L/R):** Not reported<br>**Ethnicity:** Asian<br>**Inclusion criteria:**<br>1. Diagnosis of SCZ based on DSM-IV-TR.<br>2. HCs were only included if they didn't have illnesses that could affect brain functions and other Axis I diagnoses.<br>**Exclusion criteria:** -
**Assessment**	**Paradigm class:** T-fMRI<br>**Equipment properties:** SMF = 1.5 T; Scanner = Signa Eclipse; Sequence = GE EPI, TE = 14.3 ms; TR = 2 s; Slice thickness = 5 mm; Matrix size = 64 x 64; FA = 90°; Volumes = -.
**Authors' conclusion**	"Patients with SCZ may have functional deficits in the mirror neuron system when attributing positive behaviors."
**Notes**	**Funding source:** This study was funded by the Korea Science and Engineering Foundation (KOSEF) grant funded by the Korean government (MOST) (No. R01-2005-000-10963-0).<br>**Conflicts of interest:** Not reported
**Methodological and reporting quality**	**Overall judgment:** Poor quality<br>**Notes:** The study population was not clearly specified and defined. The sample size was less than the specified minimum. The definitions, inclusion, and exclusion criteria used to identify or select cases and controls were probably not valid and reliable. Cases were not clearly defined and differentiated from controls. We cannot determine if controls were matched with cases for age and gender. We cannot determine if controls were matched with cases for handedness.

Park 2021 (23)

**Methods**	**Setting:** Seoul Youth Clinic of Seoul National University Hospital (SNUH)<br>**Country:** South Korea<br>**Ethics:** Written informed consent was obtained from all subjects after they were provided with a thorough explanation of the study procedure in the previous prospective cohort study (Institutional Review Board (IRB) no. H- 1110-009-380). In the case of minors, their parents provided written informed consent while they provided written informed assent. The study was conducted in accordance with the Declaration of Helsinki and was approved by the IRB of the SNUH (IRB no. H-2104-223-1216).
**Participants**	**Sample size:** SCZ 37; HC 80<br>**Age (Mean ± SD):** SCZ 23.05 ± 5.64; HC 22.99 ± 4.76<br>**Gender (F/M):** SCZ 21/16; HC 32/48<br>**Handedness (L/R):** SCZ 5/32; HC 5/75<br>**Ethnicity:** Asian<br>**Inclusion criteria:**<br>1. Diagnosis of SCZ, schizoaffective disorder, or schizophreniform disorder based on the Structured Clinical Interview for DSM-IV by experienced psychiatrists.<br>2. Age of 15 to 40 years.<br>3. Symptom presence for less than 2 years.<br>**Exclusion criteria:**<br>1. Past or current SCID-NP axis I diagnoses.<br>2. First- to third-degree biological relations with psychotic disorders.<br>3. History of neurological disorders and clinically severe head trauma. 4. History of substance use disorder (except nicotine).<br>5. IQ<70.<br>6. Structural and functional MRI data with intact cerebella.
**Assessment**	**Paradigm class:** rs-fMRI<br>**Equipment properties:** SMF = 3 T; Scanner = Siemens Trio scanner; Sequence = GE EPI, TE = 30ms; TR = 3.5 s; Slice thickness = 3.5 mm; Matrix size = -; FA = 90; Volumes = 35.
**Authors' conclusion**	"FEP patients, compared to HCs, displayed hyperconnectivity between the right crus I and the supplementary motor area (SMA), the left central operculum/precentral gyrus, the right precentral gyrus, and the right central/parietal operculum (CO/PO). Compared to HCs, FEP patients also showed hypoconnectivity between the right crus I and the right crus II. In addition, FEP patients, compared to HCs, showed hypoconnectivity between the right crus II and left supramarginal gyrus (SMG) and hyperconnectivity between the right crus I and left postcentral gyrus and between the right crus II and anterior cingulate gyrus (ACG) at a trend level."
**Notes**	**Funding source:** This work was supported by the Brain Research Program and the Basic Science Research Program through the National Research Foundation of Korea (NRF) and the Korea Brain Research Institute (KBRI) basic research program through the KBRI, funded by the Ministry of Science & ICT (grant nos. 2017M3C7A1029610, 2019R1C1C1002457, 2020M3E5D9079910, and 21-BR-03-01).<br>**Conflicts of interest:** No potential conflict of interest was reported by the authors.
**Methodological and reporting quality**	**Overall judgment:** Good quality<br>**Notes:** Controls were not matched with cases for gender.

Quintana 2001 (24)

**Methods**	**Setting:** Outpatients<br>**Country:** USA<br>**Ethics:** The subjects received a detailed explanation of the nature of the study and were entered in the protocol, approved by the UCLA School of Medicine and the West Los Angeles VA Health Care Center Institutional Review Boards in compliance with the Declaration of Helsinki and the guidelines from the National Institutes of Health after their informed consent was obtained.
**Participants**	**Sample size:** SCZ 8; HC 8<br>**Age (Mean ± SD):** SCZ 35.22 ± 10.69; HC 29.25 ± 5.13<br>**Gender (F/M):** SCZ 2/6; HC 2/6<br>**Handedness (L/R):** SCZ 0/8; HC 0/8<br>**Ethnicity:** Not reported<br>**Inclusion criteria:**<br>1. Diagnosis of SCZ based on DSM-IV.<br>2. HCs were only included if they didn't have a personal or family history of psychiatric or neurological disorder.<br>**Exclusion criteria:** -
**Assessment**	**Paradigm class:** T-fMRI<br>**Equipment properties:** SMF = 3 T; Scanner = -; Sequence = GE EPI, TE = 45 ms; TR = 2 s; Slice thickness = 4 mm; Matrix size = -; FA = -; Volumes = -.
**Authors' conclusion**	"We found that, when the task cues were facial expressions in contrast to color circles, the schizophrenic group exhibited increased activation of the face movement areas in motor and premotor cortex."
**Notes**	**Funding source:** Supported by a Department of Veteran Affairs (Office of Research and Development, Medical Research Service) Advanced Research Scientist Career Development Award, and by a NARSAD Staglin Young Investigator Award to J.Q., as well as by contributions from the VA's VISN-22 MIRECC, the Brain Mapping Medical Research Organization, the Brain Mapping Support Foundation, the Pierson-Lovelace Foundation, The Ahmanson Foundation, the Tamkin Foundation, the Jennifer-Jones Simon Foundation, the Capital Group Companies Charitable Foundation, the Robson Family, the Northstar Fund, and a National Center for Research Resources grant RR12169.<br>**Conflicts of interest:** Not reported
**Methodological and reporting quality**	**Overall judgment:** Poor quality<br>**Notes:** The sample size was less than the specified minimum. The definitions, inclusion, and exclusion criteria used to identify or select cases and controls were probably not valid and reliable. Controls were not matched with cases for age. We cannot determine if the measures of the assessment paradigm are valid and reliable for this review's subject.

Saito 2018 (25)

**Methods**	**Setting:** Hospital and outpatient clinics, referrals from clinicians, advertisements, and outreach presentations<br><br>**Country:** USA<br><br>**Ethics:** This study was approved by the local institutional review board. Written informed consent was obtained for all subjects.
**Participants**	**Sample size:** SCZ 16; HC 16<br><br>**Age (Mean ± SD):** SCZ 20.19 ± 4.51; HC 21.88 ± 2.71<br><br>**Gender (F/M):** SCZ 4/12; HC 6/10<br><br>**Handedness (L/R):** Matched<br><br>**Ethnicity:** 16 White, 5 African American, 2 Asian, 9 Unknown<br><br>**Inclusion criteria:**<br>1. Diagnosis of SCZ based on Structured Clinical Interview for the DSM-IV-TR.<br>2. HCs were only included if they didn't have any history of psychiatric hospitalizations, prodromal symptoms, schizotypal or other Cluster A personality disorders, first-degree relatives with psychosis, or any current or past use of antipsychotic.<br><br>**Exclusion criteria:**<br>1. History of psychosis, Major Depression (recurrent), Bipolar disorder, Obsessive Compulsive Disorder, Post Traumatic Stress Disorder, developmental disorders, neurological disorders, neurocognitive dysfunction, mental retardation, education <9th grade, not fluent in English, substance abuse in the past month, substance dependence, excluding nicotine in the past 3 months, current suicidality, ECT within the past 5 years for SCZs and ever for HCs.
**Assessment**	**Paradigm class:** DWI/DTI<br><br>**Equipment properties:** SMF = 3 T; Scanner = Echospeed system; Sequence = GE, TE = 80 ms; TR = 17 s; Slice thickness = 1.7 mm; Matrix size = 144 x 144.
**Authors' conclusion**	"We have demonstrated disrupted white matter microstructure within the white matter tracts subserving brain regions containing mirror neurons."
**Notes**	**Funding source:** This work was supported in part by a Department of Veteran Affairs Merit Award (MES), and in part by NIH grants including 1P50MH080272 CIDAR (RWM, MES), P41 RR13218 (MES), R01 MH 50740 (MES), NA-MIC (NIH) grant U54 GM072977 (MK), 1R01 AG04252 (MK and OP), R01 MH102377 (MK, MES, and OP). R01 MH074794 (OP), 2P41 EB015902-16 (OP, MES), the Commonwealth Research Center (SCDMH82101008006; LJS), NARSAD (ZK and OP), Else Kröner-Fresenius Stiftung, Germany (IK), NIMH R21MH094509 (MAN).<br><br>**Conflicts of interest:** The Authors Yukiko Saito, Marek Kubicki, Inga Koerte, Tatsui Otsuka, Yogesh Rathi, Ofer Pasternak, Sylvain Bouix, Ryan Eckbo, Zora Kikinis, Christian Clemm von Hohenberg, Tomohide Roppongi, Elisabetta Del Re, Takeshi Asami, Sang-Hyuk Lee, Raquelle I. Mesholam-Gately, Larry J. Seidman, James Levitt, Robert W. McCarley, Martha E. Shenton, and Margaret A. Niznikiewicz have declared that there are no conflicts of interest concerning the subject of this study.
**Methodological and reporting quality**	**Overall judgment:** Good quality<br><br>**Notes:** The sample size was less than the specified minimum.

Schilbach 2016 (26)

**Methods**	**Setting:** 5 sites: Aachen, Albuquerque, Göttingen, Groningen, and Utrecht<br>**Country:** Germany<br>**Ethics:** All participants gave written informed consent to participate in the study as approved by the ethics committees of the universities of Aachen, Albuquerque, Goettingen, Utrecht, and Groningen. Joint re-analysis was approved by the ethics committee of the Heinrich-Heine University Duesseldorf.
**Participants**	**Sample size:** SCZ 116; HC 133<br>**Age (Mean ± SD):** SCZ 34.25 ± 11.58; HC 34.53 ± 11.91<br>**Gender (F/M):** SCZ 31/87; HC 41/92<br>**Handedness (L/R):** Not reported<br>**Ethnicity:** White<br>**Inclusion criteria:**<br>1. HCs were only included if they didn't have any history of any psychiatric or neurological disorder, confirmed via structured clinical interview (SCID).<br>2. Diagnosis of SCZ based on ICD-10 or DSM-IV-TR by the attending psychiatrist.<br>**Exclusion criteria:** -
**Assessment**	**Paradigm class:** rs-fMRI<br>**Equipment properties:** SMF: 3T, 3T, 3T, 3T, 3T; Scanner: Siemens Tim Trio, Philips Achieva, Philips Achieva, Siemens Tim Trio, Siemens Tim Trio; Sequence: GE EPI, GE EPI, PRESTO-SENSE, GE EPI, GE EPI; TE: 28 ms, 28 ms, 32.4 ms, 30 ms, 29 ms; TR: 2 s, 2.4 s, 21.75 ms, 2 s, 2 s; Slice Thickness: 3.3 mm, 3 mm, 4 mm, 3 mm, 3.5 mm; FA: 77, 85, 10, 70, 75; Volumes: 210, 200, 600, 156, 150.
**Authors' conclusion**	"Results demonstrated decreased connectivity within the mirror neuron system and also the mentalizing network in patients compared to controls."
**Notes**	**Funding source:** L.S. was supported by the Volkswagen Foundation, the Deutsche Forschungsgemeinschaft (DFG), and the Max- Planck Society (MPG). B.D. was supported by JARA BRAIN and the DFG (DE 2319/2-3). S.B.E. was supported by the DFG (EI 816/4-1, LA 3071/3-1; EI 816/6-1), the National Institute of Mental Health (R01-MH074457), the Helmholtz Portfolio Theme "Supercomputing and Modeling for the Human Brain" and the European Union Seventh Framework Programme (FP7/2007–2013) under grant agreement no. 604102 (Human Brain Project).<br>**Conflicts of interest:** No potential conflict of interest was reported by the authors.
**Methodological and reporting quality**	**Overall judgment:** Fair quality<br>**Notes:** The definitions, inclusion, and exclusion criteria used to identify or select cases and controls were probably not valid and reliable. We cannot determine if controls were matched with cases for handedness.

Schurmann 2007 (27)

**Methods**	**Setting:** Outpatients<br>**Country:** Finland<br>**Ethics:** Informed consent and ethics committee approval.
**Participants**	**Sample size:** SCZ 11; HC 11<br>**Age (Mean ± SD):** SCZ 54.4 ± 4.8; HC 54.4 ± 4.8<br>**Gender (F/M):** Matched<br>**Handedness (L/R):** Not reported<br>**Ethnicity:** White<br>**Inclusion criteria:**<br>1. Diagnosis of SCZ based on Structural Clinical Interview for DSM-II-R.<br>**Exclusion criteria:**<br>1. A diagnosis of schizoaffective disorder, affective type in a twin with the manifest disease, or a psychotic disorder diagnosis in a non-affected twin.
**Assessment**	**Paradigm class:** MEG<br>**Equipment properties:** Signals were collected from 204 planar gradiometers of a helmet-shaped whole-scalp neuromagnetometer (Vectorview).
**Authors' conclusion**	"During action observation and execution, motor cortex reaction was weaker in those with schizophrenia than in their co-twins, suggesting a disease-related dysfunction of motor cognition."
**Notes**	**Funding source:** Supported by the Academy of Finland (National Centers of Excellence Programme 2006-2011), Sigrid Juselius Foundation, and the National Institute of Mental Health, USA (MH52857).<br>**Conflicts of interest:** No potential conflict of interest was reported by the authors.
**Methodological and reporting quality**	**Overall judgment:** Poor quality<br>**Notes:** The sample size was less than the specified minimum. The definitions, inclusion, and exclusion criteria used to identify or select cases and controls were probably not valid and reliable. We cannot determine if controls were matched with cases for handedness.

Singh 2011 (28)

**Methods**	**Setting:** The UCSD Outpatient Psychiatric services clinic<br>**Country:** USA<br>**Ethics:** This project was reviewed and approved by the UCSD Human Research Protections Program.
**Participants**	**Sample size:** SCZ 20 (all first-episode psychosis); HC 12<br>**Age (Mean ± SD):** SCZ 19.15 ± 4.3; HC 23.75 ± 5.8<br>**Gender (F/M):** SCZ 4/16; HC 6/6<br>**Handedness (L/R):** SCZ 2/18; HC 1/11<br>**Ethnicity:** Not reported<br>**Inclusion criteria:**<br>1. The first episode of psychosis within the last 2 years, based on DSM-IV.<br>2. Normal or corrected-to-normal vision.<br>**Exclusion criteria:**<br>1. History of a traumatic brain injury or seizure disorder.<br>2. History of substance abuse or dependence in the last month per history or urine toxicology screen.
**Assessment**	**Paradigm class:** EEG<br>**Equipment properties:** Device: Neuroscan Synamps 4.2 system; SR: 500 Hz; Channels: -; Bandpass filter: 0.1-30 Hz; Cap: -; System: International 10-20; EOG: -.
**Authors' conclusion**	"Compared to typically developing individuals, first-episode subjects showed similar mu wave suppression when viewing 1) a moving human hand and 2) a social interaction depicted by a game of catch. FE subjects showed significantly lower mu wave suppression when viewing biological motion in a point-light display animation video."
**Notes**	**Funding source:** Funding for this study was provided by the Mental Illness Research, Education and Clinical Center (MIRECC) program, Academic Senate Grant from the University of California at San Diego, and the National Institute of Mental Health (R01 MH060720, K24 MH076191, and MH076191).<br>**Conflicts of interest:** No potential conflict of interest was reported by the authors.
**Methodological and reporting quality**	**Overall judgment:** Poor quality<br>**Notes:** We cannot determine if the research question or objective is appropriate for this review's subject. The sample size was less than the specified minimum. Controls were not matched with cases for age and gender.

Stegmayer 2018 (29)

**Methods**	**Setting:** Inpatient and outpatient departments of the University Hospital of Psychiatry, Bern<br>**Country:** Switzerland<br>**Ethics:** All participants provided written informed consent. The study protocol adhered to the declaration of Helsinki and was approved by the local Ethics Committee.
**Participants**	**Sample size:** SCZ 22 (16 SCZ, 4 schizophreniform, 2 schizoaffective); HC 25<br>**Age (Mean ± SD):** SCZ 37.5 ± 9.8; HC 39.2 ± 14.0<br>**Gender (F/M):** SCZ 8/14; HC 12/13<br>**Handedness (L/R):** SCZ 0/22; HC 0/25<br>**Ethnicity:** White<br>**Inclusion criteria:**<br>1. Diagnosis of SCZ based on DSM-V.<br>2. Right-handedness.<br>3. HCs were only included if they didn't have any history of any psychiatric disorder, as well as any first-degree relatives with SCZ or schizoaffective disorder.<br>**Exclusion criteria:**<br>1. History of substance abuse or dependence other than nicotine.<br>2. History of motor impairments such as dystonia, idiopathic parkinsonism, or stroke.<br>3. History of head trauma with concurrent loss of consciousness.<br>4. History of electroconvulsive treatment.
**Assessment**	**Paradigm class:** T-fMRI<br>**Equipment properties:** SMF = 3 T; Scanner = Siemens Magnetom Trio; Sequence = GE EPI, TE = 40 ms; TR = 2 s; Slice thickness = 3 mm; Matrix size = 64 x 64; FA = 16°; Volumes = 390.
**Authors' conclusion**	"Our results substantiate earlier findings demonstrating aberrant mirror neuron activation within the IPL during both action observation and action execution in SCZ."
**Notes**	**Funding source:** This work was supported by the Bangerter-Rhyner Foundation (to S.W.) and the Swiss National Science Foundation (SNF grant 152619/1 to S.W., A.F., and S.B.).<br>**Conflicts of interest:** No potential conflict of interest was reported by the authors.
**Methodological and reporting quality**	**Overall judgment:** Good quality<br>**Notes:** Controls were not matched with cases for gender.

Sun 2021 (30)

**Methods**	**Setting:** Hospital of Zhejiang University<br>**Country:** China<br>**Ethics:** Written consent was obtained from all participants or their parents and legal guardians. The local ethics committee approved the IRB protocol.
**Participants**	**Sample size:** SCZ 28 (first-episode, drug-naive adolescent-onset schizophrenia); HC 22<br>**Age (Mean ± SD):** SCZ 16.8 ± 1.2; HC 16.3 ± 2.3<br>**Gender (F/M):** SCZ 13/15; HC 12/10<br>**Handedness (L/R):** SCZ 0/28; HC 0/22<br>**Ethnicity:** Asian<br>**Inclusion criteria:**<br>1. Diagnosis of SCZ based on DSM-IV.<br>2. Age of 13-18 years.<br>3. Having onset of a first psychotic episode within 24 months of program entry.<br>4. Antipsychotic drug-naive.<br>5. BMI ≥18.0 kg/m^2 and < 30 kg/m^2.<br>**Exclusion criteria:**<br>1. History or presence of any severe unstable general medical illness.<br>2. History of neurological brain disease, cerebral trauma, seizure disorder, mental retardation, or MRI evidence of structural brain abnormalities.<br>3. Pregnancy, breastfeeding, any ongoing family planning.<br>4. Any contraindication or incompatibility for MRI.
**Assessment**	**Paradigm class:** rs-fMRI<br>**Equipment properties:** SMF = 3 T; Scanner = Philips Achieva; Sequence = GE EPI, TE = 35 ms; TR = 2 s; Slice thickness = 5 mm; Matrix size = 100 x 100; FA = 80°; Volumes = 200.
**Authors' conclusion**	"Adolescent-onset schizophrenia (AOS) patients showed several state-specific connectivity decreases within the mirror neuron system (MNS)."
**Notes**	**Funding source:** This work was supported in part by grants sponsored by China National Key R&D Program [No. 2016YFC1307005/ 2017YFC1308502 / 2017YFC1308500]; a grant sponsored by China National Science Foundation [No.81471734]; a grant of Basic Public Welfare Research Projects in Zhejiang Province [No. LGF18H090003]; and a grant of Major Subjects of Zhejiang Province [No. 2015C03054]; and a grant by the Shanghai Commission of Science and Technology [No. 10550720500].<br>**Conflicts of interest:** No potential conflict of interest was reported by the authors.
**Methodological and reporting quality**	**Overall judgment:** Good quality<br>**Notes:** The definitions, inclusion, and exclusion criteria used to identify or select cases and controls were probably not valid and reliable (did not evaluate substance abuse/dependence).

Thakkar 2014 (31)

**Methods**	**Setting:** Psychiatric facility in Nashville, Tenn<br>**Country:** USA<br>**Ethics:** Participants gave written informed consent, as approved by the Vanderbilt Institutional Review Board, and they received compensation for their participation.
**Participants**	**Sample size:** SCZ 16; HC 16<br>**Age (Mean ± SD):** SCZ 40.2 ± 9.1; HC 37.4 ± 7.0<br>**Gender (F/M):** Not reported<br>**Handedness (L/R):** Not reported<br>**Ethnicity:** Not reported<br>**Inclusion criteria:**<br>1. Diagnosis of SCZ based on the Structured Clinical Interview for DSM-IV.<br>2. HCs were only included if they didn't have any history of DSM-IV axis I disorders.<br>3. Normal or corrected-to-normal vision.<br>**Exclusion criteria:**<br>1. Substance use or alcohol abuse within the past 6 months.<br>2. Brain injury.<br>3. Neurological disease.
**Assessment**	**Paradigm class:** T-fMRI<br>**Equipment properties:** SMF = 3 T; Scanner = Philips Intera Achieva; Sequence = GE EPI, TE = 35 ms; TR = 2 s; Slice thickness = 3 mm; Matrix size = 64 x 64; FA = 79°; Volumes = -.
**Authors' conclusion**	"We observed abnormal activity in the mirror neuron system in SCZ. In patients, activation in the posterior superior temporal sulcus and inferior parietal lobe was less specific to action imitation compared with non-imitative action. Furthermore, activity in these two regions was reduced during action observation in SCZ. Given the specific role of these regions in social cognition, the impaired imitative ability may be rooted in a faulty perception of biological motion and erroneous transformation of visual information into motor representations."
**Notes**	**Funding source:** Supported by NIH grants R01-MH073028 to Dr. Park and F31-MH085405-01 to Dr. Thakkar; a NARSAD Distinguished Investigator Award to Dr. Park; a Rubicon grant from the Netherlands Organization for Scientific Research to Dr. Thakkar; and grant UL1 RR024975-01 from the National Center for Research Resources.<br>**Conflicts of interest:** No potential conflict of interest was reported by the authors.
**Methodological and reporting quality**	**Overall judgment:** Poor quality<br>**Notes:** The sample size was less than the specified minimum. Cases were not clearly defined and differentiated from controls. We cannot determine if controls were matched with cases for age and gender. We cannot determine if controls were matched with cases for handedness.

Tseng 2015 (32)

**Methods**	**Setting:** National Taiwan University Hospital<br>**Country:** Taiwan<br>**Ethics:** This study was approved by the Institutional Review Board of the hospital and written informed consent was obtained from the participants.
**Participants**	**Sample size:** SCZ 32; HC 32<br>**Age (Mean ± SD):** SCZ 32.4 ± 6.3; HC 32.0 ± 6.5<br>**Gender (F/M):** SCZ 15/17; HC 15/17<br>**Handedness (L/R):** SCZ 0/32; HC 0/32<br>**Ethnicity:** Asian<br>**Inclusion criteria:**<br>1. Diagnosis of SCZ based on DSM-IV.<br>2. Right handedness.<br>3. HCs were only included if they didn't have schizophrenia, other psychiatric disorders, or a history of neurological diseases.<br>**Exclusion criteria:**<br>1. Presence of DSM-IV Axis I diagnoses of other disorders or a history of neurological diseases.
**Assessment**	**Paradigm class:** DSI<br>**Equipment properties:** Scanner = -; Sequence = SE EPI, TE = 130 ms; TR = 9.6 s; Slice thickness = 2.5 mm; Matrix size = 80 x 80; b_{max} = 4000 s/mm^2; Volumes = 102.
**Authors' conclusion**	"We found that cortical thickness and fiber tract integrity of the MNS were generally reduced, with a significant reduction in bilateral SMg, bilateral Pop, and CC-Pop."
**Notes**	**Funding source:** This work was supported in part by the Ministry of Science and Technology (NSC100-3112-B-002-016, MOST-103-2325-B-002-040), the National Health Research Institute (NHRI-EX103-10145NI), and the Ministry of Economic Affairs (Grant number: 101-EC-17-A-19-S1-175).<br>**Conflicts of interest:** Not reported
**Methodological and reporting quality**	**Overall judgment:** Good quality<br>**Notes:** The definitions, inclusion, and exclusion criteria used to identify or select cases and controls were probably not valid and reliable (did not evaluate substance abuse/dependence).

Varcin 2010 (33)

**Methods**	**Setting:** Outpatient clinics in Sydney<br>**Country:** Australia<br>**Ethics:** All testing procedures received ethical approval from the South Eastern Sydney Area Health Service in conjunction with the University of New South Wales.
**Participants**	**Sample size:** SCZ 25 (15 SCZ, 10 schizoaffective); HC 25<br>**Age (Mean ± SD):** SCZ 42.9 ± 9.43; HC 39.2 ± 10.58<br>**Gender (F/M):** SCZ 15/10; HC 14/11<br>**Handedness (L/R):** Not reported<br>**Ethnicity:** White<br>**Inclusion criteria:**<br>1. Diagnosis of SCZ based on DSM-IV.<br>2. Age over 18.<br>3. HCs were only included if they didn't have any history of psychopathology.<br>**Exclusion criteria:**<br>1. Neurological disorder.<br>2. Presence of motor abnormalities.<br>3. Alcohol/drug abuse<br>4. Any sensory impairment that would interfere with testing.
**Assessment**	**Paradigm class:** EMG<br>**Equipment properties:** Device: PowerLab 8/30 Data Acquisition System; SR: 2000 Hz; Channels: 5; Bandpass filter: 10-500 Hz; Amplification factor: 2000.
**Authors' conclusion**	"Zygomaticus muscle activity was lower in the schizophrenia group when viewing facial stimuli depicting happiness. Corrugator muscle activity was lower in the schizophrenia group when viewing facial stimuli depicting anger."
**Notes**	**Funding source:** This research was supported by an Australian Research Council Discovery Grant.<br>**Conflicts of interest:** Not reported
**Methodological and reporting quality**	**Overall judgment:** Poor quality<br>**Notes:** We cannot determine if the research question or objective is appropriate for this review's subject. We cannot determine if controls were matched with cases for handedness. We cannot determine if the measures of the assessment paradigm are valid and reliable for this review's subject.

Zaytseva 2017 (34)

**Methods**	**Setting:** Not reported<br>**Country:** Russia<br>**Ethics:** Not reported
**Participants**	**Sample size:** SCZ 11 (2 catatonic, 9 paranoid); HC 32<br>**Age (Mean ± SD):** SCZ 22.1 ±1.2; HC 23.0 ± 0.8<br>**Gender (F/M):** SCZ 0/11; HC 0/32<br>**Handedness (L/R):** Not reported<br>**Ethnicity:** White<br>**Inclusion criteria:**<br>1. Diagnosis of SCZ based on ICD-10.<br>**Exclusion criteria:** -
**Assessment**	**Paradigm class:** EEG<br>**Equipment properties:** Device: -; SR: -; Channels: 19; Bandpass filter: 6-14 Hz; Cap: -; System: International 10-20; EOG: -.
**Authors' conclusion**	"A lack of mu rhythm suppression indicates dysfunction in the parietal and frontal cortices, corresponding to decreased motor control and programming of voluntary movements as well as indirectly pointing to alterations in the mirror neurons system."
**Notes**	**Funding source:** This project was supported by the Russian Foundation for Humanities (14-06-00304).<br>**Conflicts of interest:** No potential conflict of interest was reported by the authors.
**Methodological and reporting quality**	**Overall judgment:** Poor quality<br>**Notes:** The research question or objective was not clearly stated. The sample size was less than the specified minimum. It was not reported if controls were screened for other psychiatric or neurological disorders. The definitions, inclusion, and exclusion criteria used to identify or select cases and controls were probably not valid and reliable. We cannot determine if controls were matched with cases for handedness. We cannot determine if the measures of the assessment paradigm are valid and reliable for this review's subject. It was not reported if ethical issues were considered in the study design.

References

1. Andereasen N, Calage C, O'Leary D. Theory of Mind and Schizophrenia: A Positron Emission Tomography Study of Medication-Free Patients. Schizophr Bull [Internet]. 2009 Sep 1;35(5):1030–1030. Available from: https://academic.oup.com/schizophreniabulletin/article-lookup/doi/10.1093/schbul/sbp069

2. Andrews SC, Enticott PG, Hoy KE, Thomson RH, Fitzgerald PB. No evidence for mirror system dysfunction in schizophrenia from a multimodal TMS/EEG study. Psychiatry Res [Internet]. 2015 Aug;228(3):431–40. Available from: https://linkinghub.elsevier.com/retrieve/pii/S0165178115003728

3. Bagewadi VI, Mehta UM, Naik SS, Govindaraj R, Varambally S, Arumugham SS, et al. Diminished modulation of motor cortical reactivity during context-based action observation in schizophrenia. Schizophr Res [Internet]. 2019 Feb;204:222–9. Available from: https://linkinghub.elsevier.com/retrieve/pii/S0920996418304894

4. Brown EC, Gonzalez-Liencres C, Tas C, Brüne M. Reward modulates the mirror neuron system in schizophrenia: A study into the mu rhythm suppression, empathy, and mental state attribution. Soc Neurosci [Internet]. 2016 Mar 3;11(2):175–86. Available from: http://www.tandfonline.com/doi/full/10.1080/17470919.2015.1053982

5. Choe E, Lee TY, Kim M, Hur JW, Yoon YB, Cho KIK, et al. Aberrant within- and between-network connectivity of the mirror neuron system network and the mentalizing network in first episode psychosis. Schizophr Res [Internet]. 2018 Sep;199:243–9. Available from: https://linkinghub.elsevier.com/retrieve/pii/S0920996418301750

6. Das P, Lagopoulos J, Coulston CM, Henderson AF, Malhi GS. Mentalizing impairment in schizophrenia: A functional MRI study. Schizophr Res [Internet]. 2012 Feb;134(2–3):158–64. Available from: https://linkinghub.elsevier.com/retrieve/pii/S0920996411004695

7. ElShahawi HH, Sakr HM, Hashim MA, Mohamed HH, Abdeen MS. Social cognition correlation to white matter integrity alteration in mirror neurons of schizophrenic patients: DTI study. Neurol Psychiatry Brain Res [Internet]. 2020 Dec;38:65–73. Available from: https://linkinghub.elsevier.com/retrieve/pii/S0941950020301445

8. Enticott P, Hoy K, Herring S, Johnson P, Daskalakis Z, Fitzgerald P. Reduced motor facilitation during action observation in schizophrenia: A mirror neuron deficit? Schizophr Res [Internet]. 2008 Jul;102(1–3):116–21. Available from: https://linkinghub.elsevier.com/retrieve/pii/S0920996408001679

9. Ferri F, Costantini M, Salone A, Ebisch S, de Berardis D, Mazzola V, et al. Binding Action and Emotion in First-Episode Schizophrenia. Psychopathology [Internet]. 2014;47(6):394–407. Available from: https://www.karger.com/Article/FullText/366133

10. Guo S, Kendrick KM, Yu R, Wang HLS, Feng J. Key functional circuitry altered in schizophrenia involves parietal regions associated with sense of self. Hum Brain Mapp [Internet]. 2014 Jan;35(1):123–39. Available from: https://onlinelibrary.wiley.com/doi/10.1002/hbm.22162

11. He Y, Steines M, Sammer G, Nagels A, Kircher T, Straube B. Modality-specific dysfunctional neural processing of social-abstract and non-social-concrete information in schizophrenia. Neuroimage Clin [Internet]. 2021;29:102568. Available from: https://linkinghub.elsevier.com/retrieve/pii/S2213158221000127

12. Horan WP, Iacoboni M, Cross KA, Korb A, Lee J, Nori P, et al. Self-reported empathy and neural activity during action imitation and observation in schizophrenia. Neuroimage Clin [Internet]. 2014;5:100–8. Available from: https://linkinghub.elsevier.com/retrieve/pii/S2213158214000825

13. Horan WP, Pineda JA, Wynn JK, Iacoboni M, Green MF. Some markers of mirroring appear intact in schizophrenia: evidence from mu suppression. Cogn Affect Behav Neurosci [Internet]. 2014 Sep 11;14(3):1049–60. Available from: https://link.springer.com/10.3758/s13415-013-0245-8

14. Horan WP, Jimenez AM, Lee J, Wynn JK, Eisenberger NI, Green MF. Pain empathy in schizophrenia: an fMRI study. Soc Cogn Affect Neurosci [Internet]. 2016 May 1;11(5):783–92. Available from: https://academic.oup.com/scan/article/11/5/783/1753435

15. Kato Y, Muramatsu T, Kato M, Shibukawa Y, Shintani M, Mimura M. Magnetoencephalography Study of Right Parietal Lobe Dysfunction of the Evoked Mirror Neuron System in Antipsychotic-Free Schizophrenia. Hashimoto K, editor. PLoS One [Internet]. 2011 Nov 22;6(11):e28087. Available from: https://dx.plos.org/10.1371/journal.pone.0028087

16. Lee JS, Chun JW, Yoon SY, Park HJ, Kim JJ. Involvement of the mirror neuron system in blunted affect in schizophrenia. Schizophr Res [Internet]. 2014 Jan;152(1):268–74. Available from: https://linkinghub.elsevier.com/retrieve/pii/S0920996413005938

17. McCormick LM, Brumm MC, Beadle JN, Paradiso S, Yamada T, Andreasen N. Mirror neuron function, psychosis, and empathy in schizophrenia. Psychiatry Res Neuroimaging [Internet]. 2012 Mar;201(3):233–9. Available from: https://linkinghub.elsevier.com/retrieve/pii/S0925492712000054

18. Mehta UM, Thirthalli J, Basavaraju R, Gangadhar BN, Pascual-Leone A. Reduced Mirror Neuron Activity in Schizophrenia and Its Association With Theory of Mind Deficits: Evidence From a Transcranial Magnetic Stimulation Study. Schizophr Bull [Internet]. 2014 Sep;40(5):1083–94. Available from: https://academic.oup.com/schizophreniabulletin/article-lookup/doi/10.1093/schbul/sbt155

19. Mitra S, Nizamie SH, Goyal N, Tikka SK. Mu-wave Activity in Schizophrenia: Evidence of a Dysfunctional Mirror Neuron System from an Indian Study. Indian J Psychol Med [Internet]. 2014 Jul 1;36(3):276–81. Available from: http://journals.sagepub.com/doi/10.4103/0253-7176.135380

20. Möhring N, Shen C, Hahn E, Ta TMT, Dettling M, Neuhaus AH. Mirror neuron deficit in schizophrenia: Evidence from repetition suppression. Schizophr Res [Internet]. 2015 Oct;168(1–2):174–9. Available from: https://linkinghub.elsevier.com/retrieve/pii/S0920996415004016

21. Okruszek Ł, Wordecha M, Jarkiewicz M, Kossowski B, Lee J, Marchewka A. Brain correlates of recognition of communicative interactions from biological motion in schizophrenia. Psychol Med [Internet]. 2018 Aug 27;48(11):1862–71. Available from: https://www.cambridge.org/core/product/identifier/S0033291717003385/type/journal_article

22. Park KM, Kim JJ, Ku J, Kim SY, Lee HR, Kim SI, et al. Neural basis of attributional style in schizophrenia. Neurosci Lett [Internet]. 2009 Jul;459(1):35–40. Available from: https://linkinghub.elsevier.com/retrieve/pii/S0304394009005758

23. Park SH, Kim T, Ha M, Moon SY, Lho SK, Kim M, et al. Intrinsic cerebellar functional connectivity of social cognition and theory of mind in first-episode psychosis patients. NPJ Schizophr [Internet]. 2021 Dec 3;7(1):59. Available from: https://www.nature.com/articles/s41537-021-00193-w

24. Quintana J. A Compensatory Mirror Cortical Mechanism for Facial Affect Processing in Schizophrenia. Neuropsychopharmacology [Internet]. 2001 Dec;25(6):915–24. Available from: http://www.nature.com/doifinder/10.1016/S0893-133X(01)00304-9

25. Saito Y, Kubicki M, Koerte I, Otsuka T, Rathi Y, Pasternak O, et al. Impaired white matter connectivity between regions containing mirror neurons, and relationship to negative symptoms and social cognition, in patients with first-episode schizophrenia. Brain Imaging Behav [Internet]. 2018 Feb 28;12(1):229–37. Available from: http://link.springer.com/10.1007/s11682-017-9685-z

26. Schilbach L, Derntl B, Aleman A, Caspers S, Clos M, Diederen KMJ, et al. Differential Patterns of Dysconnectivity in Mirror Neuron and Mentalizing Networks in Schizophrenia. Schizophr Bull [Internet]. 2016 Sep;42(5):1135–48. Available from: https://academic.oup.com/schizophreniabulletin/article-lookup/doi/10.1093/schbul/sbw015

27. Schürmann M, Järveläinen J, Avikainen S, Cannon TD, Lönnqvist J, Huttunen M, et al. Manifest disease and motor cortex reactivity in twins discordant for schizophrenia. British Journal of Psychiatry [Internet]. 2007 Aug 2;191(2):178–9. Available from: https://www.cambridge.org/core/product/identifier/S0007125000173155/type/journal_article

28. Singh F, Pineda J, Cadenhead KS. Association of impaired EEG mu wave suppression, negative symptoms and social functioning in biological motion processing in first episode of psychosis. Schizophr Res [Internet]. 2011 Aug;130(1–3):182–6. Available from: https://linkinghub.elsevier.com/retrieve/pii/S0920996411001861

29. Stegmayer K, Bohlhalter S, Vanbellingen T, Federspiel A, Wiest R, Müri RM, et al. Limbic Interference During Social Action Planning in Schizophrenia. Schizophr Bull [Internet]. 2018 Feb 15;44(2):359–68. Available from: https://academic.oup.com/schizophreniabulletin/article/44/2/359/3858242

30. Sun F, Zhao Z, Lan M, Xu Y, Huang M, Xu D. Abnormal dynamic functional network connectivity of the mirror neuron system network and the mentalizing network in patients with adolescent-onset, first-episode, drug-naïve schizophrenia. Neurosci Res [Internet]. 2021 Jan;162:63–70. Available from: https://linkinghub.elsevier.com/retrieve/pii/S0168010219304390

31. Thakkar KN, Peterman JS, Park S. Altered Brain Activation During Action Imitation and Observation in Schizophrenia: A Translational Approach to Investigating Social Dysfunction in Schizophrenia. American Journal of Psychiatry [Internet]. 2014 May;171(5):539–48. Available from: http://psychiatryonline.org/doi/abs/10.1176/appi.ajp.2013.13040498

32. Tseng CEJ, Chien YL, Liu CM, Wang HLS, Hwu HG, Tseng WYI. Altered cortical structures and tract integrity of the mirror neuron system in association with symptoms of schizophrenia. Psychiatry Res Neuroimaging [Internet]. 2015 Mar;231(3):286–91. Available from: https://linkinghub.elsevier.com/retrieve/pii/S0925492715000116

33. Varcin KJ, Bailey PE, Henry JD. Empathic deficits in schizophrenia: The potential role of rapid facial mimicry. Journal of the International Neuropsychological Society [Internet]. 2010 Jul 7;16(4):621–9. Available from: https://www.cambridge.org/core/product/identifier/S1355617710000329/type/journal_article

34. Zaytseva Y, Morozova A, Bendova M, Garakh Z. Is motor imagery different in catatonic schizophrenia? Psych J [Internet]. 2017 Jun;6(2):137–8. Available from: https://onlinelibrary.wiley.com/doi/10.1002/pchj.155
